# How interacting pathways are regulated by miRNAs in breast cancer subtypes

**DOI:** 10.1186/s12859-016-1196-1

**Published:** 2016-11-08

**Authors:** Claudia Cava, Antonio Colaprico, Gloria Bertoli, Gianluca Bontempi, Giancarlo Mauri, Isabella Castiglioni

**Affiliations:** 1 Institute of Molecular Bioimaging and Physiology (IBFM), National Research Council (CNR), Milan, Italy; 2 Interuniversity Institute of Bioinformatics in Brussels (IB), Brussels, Belgium; 3 Machine Learning Group, ULB, Brussels, Belgium; 4 Department of Informatics, Systems and Communications, University of Milan–Bicocca, Milan, Italy

**Keywords:** miRNAs, Pathway cross-talk, Breast cancer, Network of pathways

## Abstract

**Background:**

An important challenge in cancer biology is to understand the complex aspects of the disease. It is increasingly evident that genes are not isolated from each other and the comprehension of how different genes are related to each other could explain biological mechanisms causing diseases. Biological pathways are important tools to reveal gene interaction and reduce the large number of genes to be studied by partitioning it into smaller paths. Furthermore, recent scientific evidence has proven that a combination of pathways, instead than a single element of the pathway or a single pathway, could be responsible for pathological changes in a cell.

**Results:**

In this paper we develop a new method that can reveal miRNAs able to regulate, in a coordinated way, networks of gene pathways. We applied the method to subtypes of breast cancer. The basic idea is the identification of pathways significantly enriched with differentially expressed genes among the different breast cancer subtypes and normal tissue. Looking at the pairs of pathways that were found to be functionally related, we created a network of dependent pathways and we focused on identifying miRNAs that could act as miRNA drivers in a coordinated regulation process.

**Conclusions:**

Our approach enables miRNAs identification that could have an important role in the development of breast cancer.

**Electronic supplementary material:**

The online version of this article (doi:10.1186/s12859-016-1196-1) contains supplementary material, which is available to authorized users.

## Background

The identification of breast cancer (BC) gene signatures based on morphology (stage and grade) and two key markers, estrogen receptor (ER) and human epidermal growth factor receptor 2 (HER2), are a challenge for current clinical practice [1–5].

However, the landscape of alterations in BC is more complex and heterogeneous. With the introduction of gene expression microarrays and next generation sequencing (NGS), additional studies on the molecular classification of BC were carried out. These have led to the identification of four molecular subtypes associated with distinct characteristics, distinct genetic mechanisms of disease and differences in patient survival [6, 7]: Luminal A, Luminal B, Triple Negative/Basal like and HER2 subtypes [6].

By expression profiling, the large majority of ER+ and/or progesterone receptor PgR+ tumours are “luminal subtypes” [8, 9]: Luminal A and Luminal B; they have a relatively good prognosis with the former being typically low grade [10, 11]. Luminal A is the most common subtype and represents 50 %–60 % of all BC and typically highly expresses regulated gene *SLC39A6* (solute carrier family 39 (zinc transporter), member 6), transcription factors *GATA3*, *FOXA1* and *XBP1*, and luminal cytokeratins *KRT8* and *KRT18* [12, 13]. Luminal B comprises 15 %–20 % of BC and has a more aggressive phenotype and lower survival rates after relapse [14–17]. It shows an increased expression of proliferation-related genes such as avian myeloblastosis viral oncogene homolog (*v-MYB*), gamma glutamyl hydrolase (*GGH*), lysosome-associated transmembrane protein 4-beta (*LAPTMB4*), nuclease sensitive element binding protein 1 (*NSEP1*) and cyclin E1 (*CCNE1*) [18].

For a successful discrimination of luminal-B tumours from luminal*-A* in clinical practice, Cheang et al. [8] suggested an immunohistochemistry proliferation marker, the Ki67 hormone receptor. The authors determined the Ki67 cut off point (14 %) that discriminates luminal-A from luminal-B tumours. However, Ki67 immunohistochemistry shows some limitations, such as low intra- and inter-laboratory reproducibility, the arbitrary selection of standard antibodies for testing, in addition to potential problems resulting from tumour heterogeneity [9].

Concerning the response to therapy of BC subtypes, Luminal B responds better to neoadjuvant chemotherapy, but is less responsive to hormonal therapy than Luminal A [9]. Potential targets in Luminal B are insulin-like growth factor 1 (*IGF-1*) signalling, fibroblast growth factor (*FGF*) signalling, Phosphoinositide 3-kinase signalling (*PI3K*) [19].

The interplay between ER and insulin-like growth factor 1 receptor (*IGF-1R*) shows a critical role in tamoxifen resistance. High circulating plasma levels of IGF-1, a ligand for IGF-1R, are detected in women with an increased risk of relapse on adjuvant tamoxifen [20].

Several studies indicate that the FGF factor, involved in angiogenesis [21, 22], and its receptor *FGFR1* are amplified in cells resistant to endocrine therapy [23, 24]. Knockdown of *FGFR1* and/or the use of a small molecule FGFR tyrosine kinase inhibitor could reverse resistance to endocrine therapy [23, 24].

Several methods to interrupt IGF-1 signalling, FGF signalling, and *PI3K* have been proposed [19, 25, 26]. Creighton et al. [27] suggested that the combined effect of endocrine therapy luminal-B BC cell lines and PI3K inhibitor could increase growth inhibition induced by the only endocrine therapy. Atzori et al. [26] developed antibodies against IGF-1R that block IGF-1 ligand-mediated activation and small-molecule inhibitors of the IGF-1R tyrosine kinase domain.

Some antibodies and small-molecule inhibitors against FGFR are currently in clinical testing, such as TKI-259 single agent, and Exemestane [27]. Agents targeting the PI3K pathway comprise rapamycin analogues or mTOR inhibitors [27].

The basal-like subtype, one of the most clinically aggressive groups among the different subtypes, represents 8 %–37 % of all BC, and is the one with highest rate of metastasis to the brain and lung [28]. It is more commonly negative for all 3 markers—ER, PgR, and HER2—the “triple-negative” phenotypic classification [16].

There are several other biomarkers associated with the basal subtype as well as putative candidates suitable for immunohistochemical screening [29–31].

An association between the basal subtype and germline mutations in the *BRCA1* gene, often termed the “caretaker of the genome”, has been well described, and it may be speculated that both inherent DNA damage–sensing processes and DNA repair mechanisms are central in the development of basal-like tumours [29–31]. However, currently, there is no specific international agreement on complementary biomarkers that can define basal-like cancers [32].

Given the lack of validated molecular targets in basal BC, conventional chemotherapy has been the only therapeutic option for women with this kind of tumour [33]. Based on *BRCA1* mutations, some studies explored the use of platinum chemotherapy agents (carboplatin, cisplatin, and others) [33]. Antiangiogenic therapies targeting *VEGF* and its receptors have emerged as promising therapies given the evidence of aberrant *VEGF* pathway activation in basal BC [34]. Moreover, small-molecule and antibody-based *EGFR* inhibitors are also explored as targeted therapies [35].

HER2-positive (HER2+) cancer represents 15–20 % of BC subtypes. HER2+ confers more aggressive biological and clinical behaviour [36]. These tumours present a high expression of the HER2 gene and of other genes associated with the HER2 pathway [37].

Currently, the treatment in advanced HER2+ BC is the combination of trastuzumab, pertuzumab, and the chemotherapy agent taxane [37]. High expression of HER2 promotes tamoxifen resistance and the addition of trastuzumab could improve the tamoxifen response [38].

This BC variety has consequences in the diverse clinical behaviour and provides critical insight for the development of personalized therapies.

However, gene expression microarrays and NGS data have many more genes than number of samples, and methods to reduce the dimension of genes in functional units, such as gene sets, pathways, and network modules, have been recently explored [39, 40]. These aggregation methods are based on the statement that the set of genes involved in the same biological processes are often collaborative in the development and progression of BC, and show an easier interpretation of the underlying biology [41, 42].

Methodologies to identify the set of genes enriched from a genetic signature whose combined expression pattern is that uniquely characteristic of a given phenotype are promising approaches.

Alterations of the interplay among pathways leading to uncontrolled cellular proliferation, survival, invasion, and metastases are hallmarks of the BC process.

The mitogen activated protein kinase (*MAPK*), phosphatidylinositol 3-kinase (PI3K), Akt and nuclear factor kappa B (*NF-kB)* are commonly de-regulated in different BC subtypes [36]. Raf/mitogen-activated and extracellular signal-regulated kinase (MEK)/extracellular signal-regulated kinase (ERK) are critical for normal human physiology, and also commonly dysregulated in several human cancers, including BC [43].

Saini et al. [43] suggested, both in vitro and in vivo*,* that PI3K/AKT/mTOR and Raf/MEK/ERK cascades are interconnected and the inhibition of one pathway can still result in a deregulation of the other. Notch signalling pathway is associated with many oncogenic signalling pathways, such as developmental signals, i.e., Wnt and Hedgehog signalling, growth and transcriptional factors, cytokines, and oncogenic kinases [44]. A review [45] examined the molecular basis of the collaboration and integration of the ER, and MAPK.

Currently, two main approaches have been proposed to identify functional units deregulated in a disease. One approach is to identify *de novo* functional units from the data. Following this approach, van Vliet proposed an unsupervised method to identify gene patterns using a score applied to a Bayes classifier [46]. Ma et al. [47] used weighted co expression networks and their module to describe the collaboration among genes.

The other main approaches used existing functional units to build prognostic or diagnostic analysis. Abraham et al. proposed features derived from pre-specified gene sets from the Molecular Signatures Database (MSigDB), and, by using a statistical test aggregating the expression levels of all genes within a set, they derived prognostic gene sets [48]. Huang et al. [40] proposed a new pathway-based de-regulation scoring matrix transforming the gene features in combination with Cox regression and L1-LASSO regularization to model survivals. A genomic model consisting of fifteen cancer relevant pathways was revealed and validated on three independent BCs.

Deregulation of signalling events in a given cancer sample is of great clinical interest in order to identify candidate drugs developed to specifically modulate upstream signalling events [49]. Recent progress in cancer biology has revealed that miRNAs are potential therapeutic targets suggesting the introduction of the miRNA mimic oligonucleotides in Phase I cancer clinical trials.

Oncogenic or tumour suppressive miRNAs have been implicated in the regulation of central cellular pathways, such as differentiation and apoptosis, across several tumour types [50], but the discovery of how a miRNA regulates its targets in tumour samples is still challenging. Recent studies revealed, for instance, that *Hsa-miR-21* is up regulated in BC [51], while *Hsa-miR-335* and *Hsa-miR-200c* have been shown to inhibit metastatic cell invasion [52].

Emerging evidence demonstrates that miRNAs play an essential role in controlling stem cell properties by regulating, for instance, epithelial to mesenchymal transition (EMT) [53]. EMT has a fundamental role in cancer cells with the loss of intracellular junctions and epithelial polarity. Several miRNAs, such as *Hsa-let-7*, *Hsa-miR-10*, *Hsa-miR-34*, *Hsa-miR-200*, and *Hsa-miR-205* are described as regulator of this process [53].

Other miRNAs have been reported to have an active role in tumour proliferation control. *Hsa-miR-92a* can promote tumour proliferation by controlling the PI3K/Akt/mTOR pathway [54]. Several other miRNAs were found to be up-regulated in BC; these include the *Hsa-miR-221/222* cluster [55], *Hsa-miR-9*, *Hsa-miR10b*, *Hsa-miR-29a*, *Hsa-miR-96*, *Hsa-miR-146a*, *Hsa-miR-181*, *Hsa-miR-373*, *Hsa-miR-375*, *Hsa-miR-520c*, and *Hsa-miR589* [56], suggesting their potential use for BC diagnosis, prognosis, and therapeutic studies [55, 56].

All these findings demonstrate the ability of miRNAs to regulate the development of malignancies modulating critical cancer-related genes and signalling pathways.

While many studies demonstrated the role of miRNA-target interactions in a single pathway, there are little evidence on the interaction of specific miRNAs with genes of different pathways.

*Hsa-miR-125*, whose expression correlates with the HER2 status [57], has been shown to be significantly down regulated in BC [58]. Experimentally, the overexpression of *Hsa-miR-125* decreases the expression level of *ERBB2* and *ERBB3*, reducing cell motility and invasiveness of numerous cancers, including BC [59]. The *Let-7* regulatory network suppresses metastasis acting on the chromatin-remodelling protein HMGA2 and the transcription factor *BACH1* [60]. Both targets promote the transcription of pro-invasive genes that regulate cell invasion and metastasis to the bone [60].

Another important miRNA in BC is *Hsa-miR-206*. It has been found to be down-regulated in ERα-positive BC, both in patient samples and BC cell lines [61], and in lymph node metastatic BC [62]. A critical role of *Hsa-miR-206* has been recently demonstrated in the regulation of the 3′ UTR of cyclin D1, inducing G1 arrest and a decrease in cell proliferation in BC cells [63], thus suggesting a potential role as a tumour suppressor. It has been also shown that *Hsa-miR-206* regulates ERα via interaction with its 3′ UTR [64], demonstrating a specific function in most aggressive types of BC.

In this work we developed a method to detect miRNAs regulating pathway interactions, based on the integration of gene expression profiles and biological pathways and miRNAs. We validated the approach in BC subtypes, obtaining, for each BC subtype, a network of pathways enriched from differentially expressed genes. We focused on the pairs of pathways able to differentiate a particular BC subtype with respect to the normal type. miRNAs significantly enriched from their gene targets in at least two pathways were found to be key regulators of interacting pathways.

## Methods

### Breast cancer subtypes

In our study we focused on four different BC subtypes: luminal A, luminal B, basal, and HER2 which we compared with normal samples (NS). We considered the expression level of mRNAs and miRNAs extracted from a TCGA BC data set. We performed a quantile analysis on TCGA miRNAs and mRNA, in order to exclude genes and miRNAs with a small variance, thus obtaining 1046 miRNAs and of 15243 genes. We then used BC matched samples miRNA-mRNA for all the subsequent analyses.

#### Luminal A vs. NS

We used 233 BC luminal A samples and 113 NS for mRNA analysis, and 233 BC luminal A samples and 87 NS for miRNA analysis.

#### Luminal B vs. NS

We used 103 BC luminal B samples and 113 NS for mRNA analysis, and 103 BC luminal A samples and 87 NS for miRNA analysis.

#### Basal vs. NS

We used 74 BC Basal samples and 113 NS for mRNA analysis, and 74 BC Basal samples and 87 NS samples for miRNA analysis.

#### HER2 vs. NS

We used from 43 BC HER2 samples and 113 NS for mRNA analysis, and 43 BC HER2 samples and 87 NS for miRNA analysis.

### Grouping and bootstrapping analysis

We performed an analysis based on several boots, with each boot consisting of four steps and working on different (randomly selected) training and testing data sets.

In order to perform bootstrapping, we implemented a classifier based on Monte Carlo cross validation, that randomly splits a part of the original data in the training data set (60 % in our case) and the rest of original data in the testing set (40 % in our case). The first, second and third step are performed on the training data set, the fourth step both on the training and testing data set.

In order to avoid problems of unbalanced classes of BC and NS, we randomly selected classes with the same number of BC and NS in both the training and testing dataset.

### Differentially expressed genes: 1st step

Differentially expressed genes between each subtype class of BC samples and class of NS were identified by statistical analysis using the function *TCGAanalyze DEA* from the package TCGAbiolinks from Bioconductor. The following parameters were used: quantile-adjusted conditional maximum likelihood, abs(log fold change) > 1, and FDR < 0.01 [65]. The obtained *p*-values were adjusted by using the Benjamin-Hochberg procedure for multiple testing correction [66].

### Pathways enriched from differentially expressed genes: 2nd step

Given 589 pathways derived from the Ingenuity Pathway Analysis (IPA) database, a pathway enrichment analysis was applied. The enrichment was evaluated using the Fisher’s Exact Test between differentially expressed genes and IPA pathways. We considered a pathway to be enriched if *p*-value was <0.01.

### Interacting pathways: 3rd step

Interactions among the enriched pathways were quantified by an interaction score (IS), defined as:

$$ \mathrm{IS} = \left|\left({\mathrm{M}}_{\mathrm{x}}-{\mathrm{M}}_{\mathrm{y}}\right)\right|/\left({\mathrm{S}}_{\mathrm{x}}+{\mathrm{S}}_{\mathrm{y}}\right) $$

where M_X_, S_X_, M_Y_ and S_Y_ represent the mean and the standard deviation of expression levels of genes in pathways X and Y, respectively. Maximum cross-talk was found for IS near 0.

For every comparison (BC subtype vs NS), we obtained a matrix of IS, with each raw corresponding to each BC sample and each column corresponding to IS related to each pair of significantly enriched pathways.

### Identification of the best pathways for breast cancer subtype classification: 4th step

For every comparison (BC subtype vs NS), for the training data set, we used the matrix of IS to classify the class of BC samples and NS. We used Random Forest algorithm (RF) from the R-package [67], setting the following parameters: number of variables randomly sampled at each split = sqrt(p), p being the number of variables in the matrix of data; and the number of trees grown = 500. In order to validate the classifier, we used a k-fold cross-validation (k = 10 except in case of HER2 vs. normal samples, given the reduced number of samples we used k = 5) obtaining Area Under the Curve (AUC).

We thus selected the 10 pairwise pathways with the best AUC in the classification of the different BC subtypes vs NS.

We finally validated the classification using the top 10 pairwise pathways and the same k-fold cross-validation on the testing data set in terms of AUC.

The four steps described above were repeated multiple times (50 bootstraps).

Specifically, each bootstrap generated from a training dataset i) 1 step: a list of differentially expressed genes, ii) 2 step: a list of pathways significantly enriched by differently expressed genes, iii) 3 step: a subtype-specific matrix of IS for each pair of pathways significantly enriched, and iii) 4 step: the top 10 pairwise pathways with the best AUC performance.

In conclusion, for each subtype and for all 50 bootstraps, we obtained the 10x50 (=500) pairwise pathways with the best AUCs., from which we selected, by ranking their frequency, the top 10 pairwise pathways.

### miRNAs regulating the top 10 pairwise pathways

Mutual Information (MI) was applied between the dataset of 1046 miRNAs and 15243 genes, providing a linking index between miRNAs and genes. MI was calculated using entropy estimates from K-nearest neighbour distances [68] with the R-package parmigene [69]. In this step we obtained a list of candidate target genes for each miRNA.

**Fig. 1:**
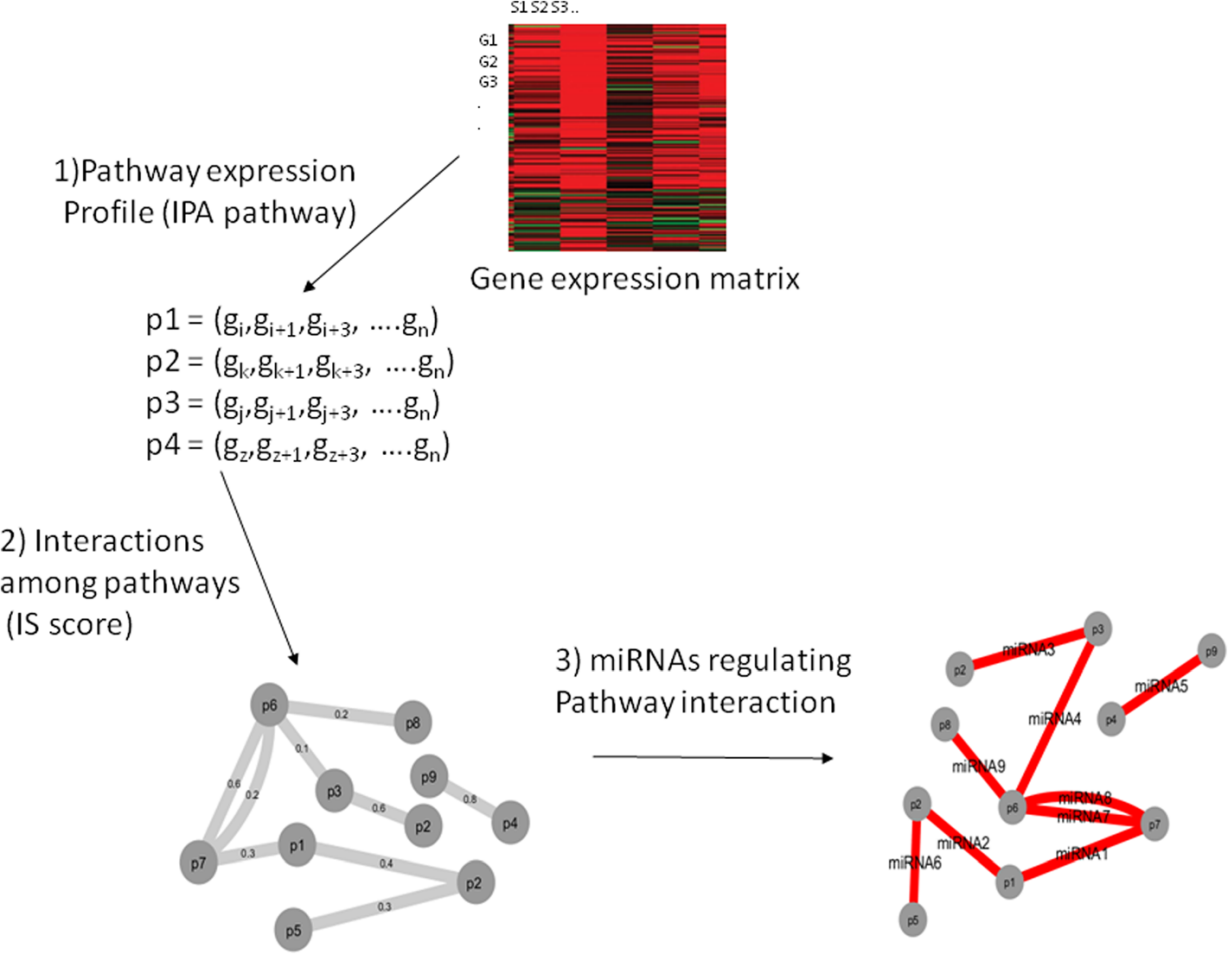
Proposed approach

In order to link miRNAs with the top 10 pairs of pathways, we applied a Fisher’s Exact Test between candidate target genes (as obtained from MI) and genes within each pairwise pathway (when *p*-value <0.01 in both pathways). We thus identified a group of miRNAs regulating the top 10 pairwise pathways (miR-r). Then, we focused only on those miR-r differentially expressed between each BC subtype and NS (quantile-adjusted conditional maximum likelihood, *p*-values adjusted using the Benjamin-Hochberg procedure for multiple testing correction [66]). Figure 1 shows the proposed procedure.

## Results

### Luminal A vs. NS

After 50 bootstraps, among the 50x10 = 500 pairwise pathways, we found 157 pairwise pathways enriched with 4703 differentially expressed genes. Indeed, many pathways were found in common among the 10 top pairs in many bootstraps.

The final top 10 pairwise pathways, selected according to their frequency in the top 10 in all bootstraps from the 157 pairwise pathways, are shown in Table 1.

**Table 1:** Luminal A: frequency of pairwise pathways in the top 10 positions for all 50 bootstraps

Pairwise pathway	Frequency
1) Ethanol Degradation IV;Glioma Invasiveness Signalling	31/50
2) Intrinsic Prothrombin Activation Pathway; Extrinsic Prothrombin Activation Pathway	28/50
3) Ethanol Degradation IV;Estrogen Receptor Signalling	25/50
4) Axonal Guidance Signalling; Acute Phase Response Signalling	17/50
5) Ethanol Degradation IV;Regulation of Cellular Mechanics by Calpain Protease	16/50
6) Glioma Invasiveness Signalling; Dopamine Degradation	15/50
7) Glioma Invasiveness Signalling; Fatty Acid oxidation	12/50
8) Glioma Invasiveness Signalling; Tryptophan Degradation X (Mammalian, via Tryptamine)	12/50
9) Acute Phase Response Signalling; HIF1 Signalling	11/50
10) Glioma Invasiveness Signalling; Oxidative Ethanol Degradation III	10/50
11) Axonal Guidance Signalling; Gs Signalling	9/50
12) HIF1 Signalling; Fatty Acid-oxidation	9/50
13) Oxidative Ethanol Degradation III; Estrogen Receptor Signalling	9/50
14) Retinoate Biosynthesis I; Estrogen Receptor Signalling	8/50
15) Tryptophan Degradation X (Mammalian, via Tryptamine); Glioma Invasiveness Signalling	8/50
….	
50) ….	1/50

Dots indicate the other pairs of pathways with minor frequency

**Fig. 2:**
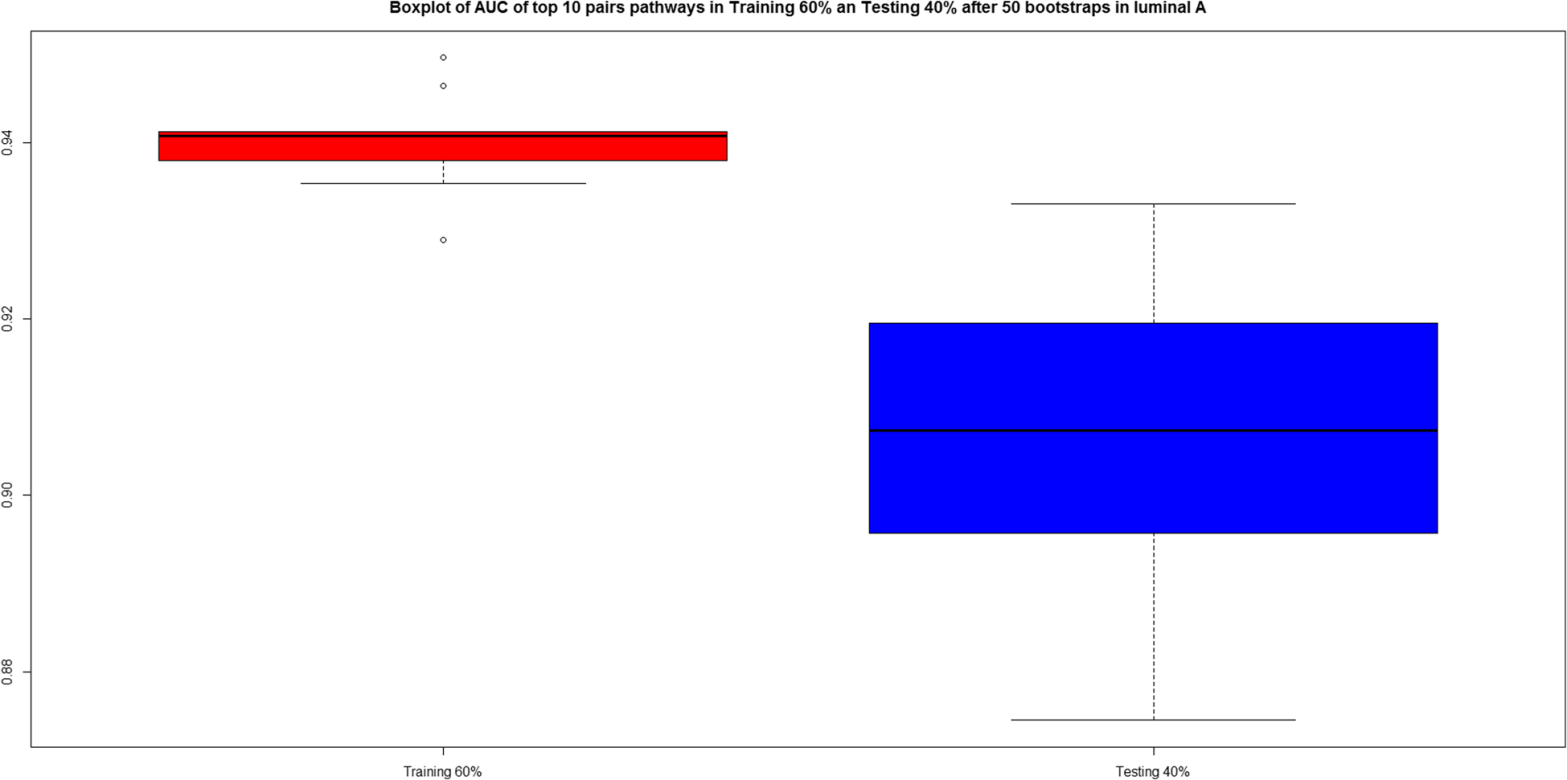
Boxplot of AUC values for the top 10 enriched pairwise pathways in luminal A, after all 50 bootstraps

Figure 2 shows a boxplot with the AUC values for the final 10 pairwise pathways in both the training and testing phase. Both AUC values are good (median >90 %), although the performance of training is better.

**Fig. 3:**
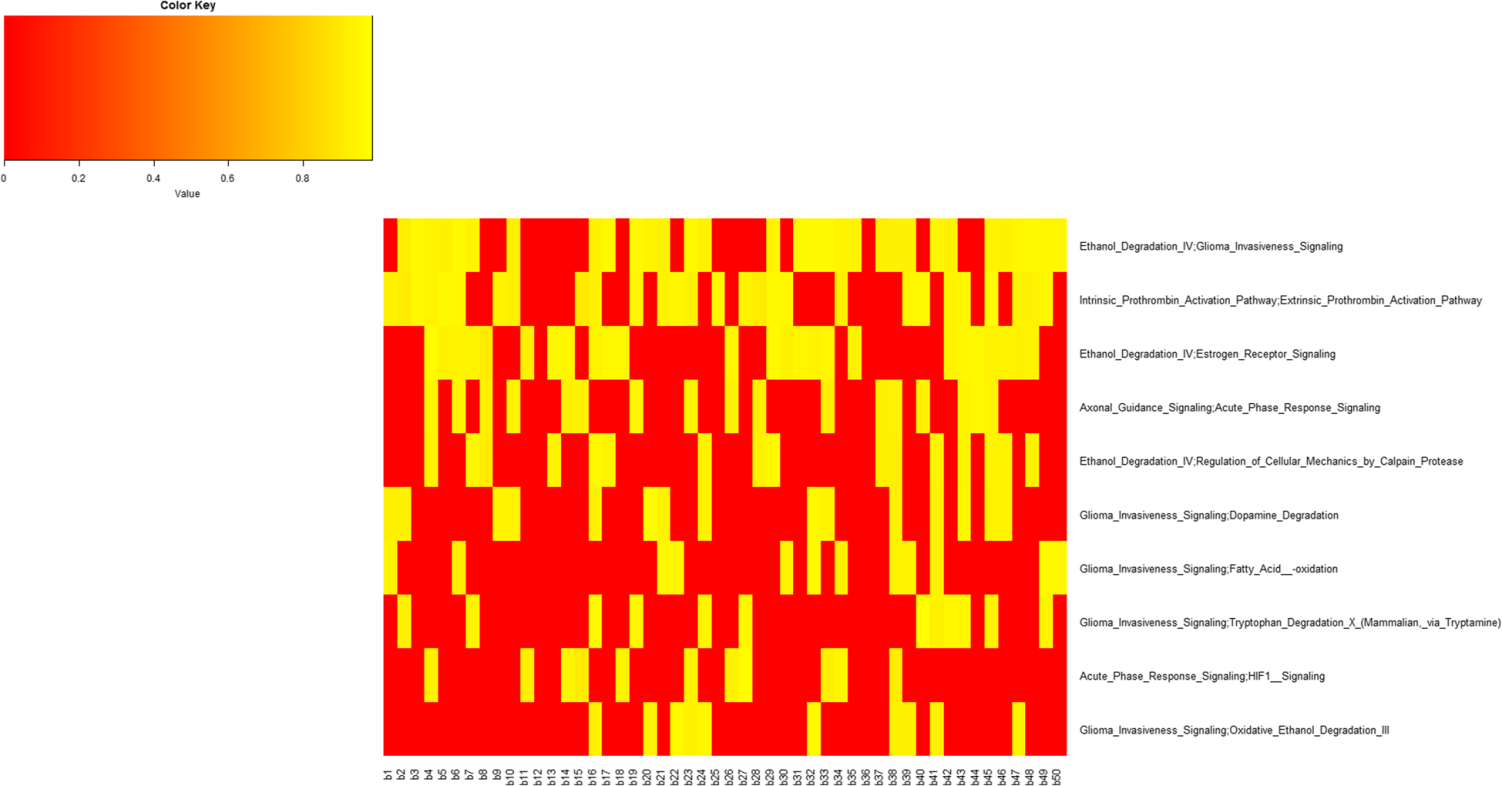
AUC values representation with the top 10 pairwise pathways for all 50 bootstraps in luminal A. *Yellow square* indicates AUC values when the pairwise pathway was included in the top 10 for the corresponding bootstrap. *Red square* indicates that the pairwise pathways was not present in top 10 for that bootstrap

Figure 3 shows, for each bootstrap, the AUC of the top 10 pairs of pathways. We can see that some pairwise pathways (e.g. Ethanol Degradation IV; Glioma Invasiveness Signalling, Intrinsic Prothrombin Activation Pathway; Extrinsic Prothrombin Activation Pathway) have excellent AUC in most bootstraps.

**Fig. 4:**
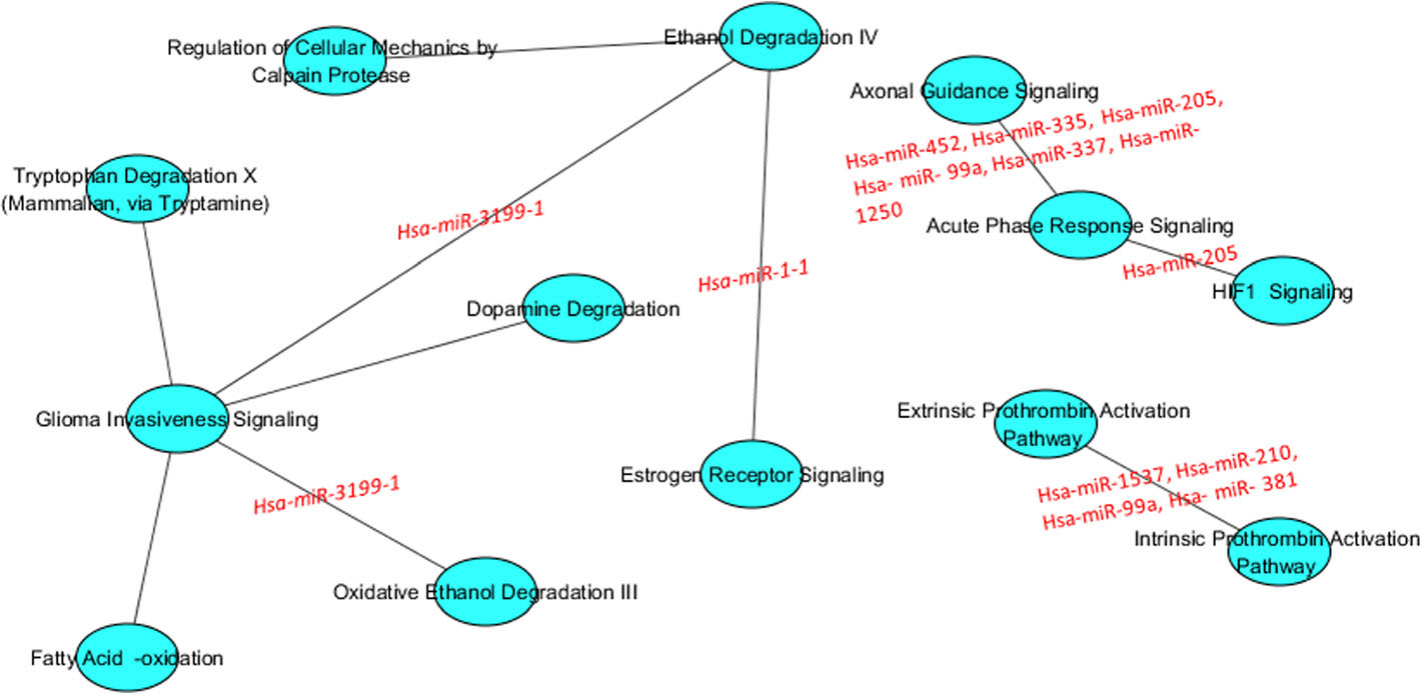
Interaction of the top 10 pairwise pathways in luminal and their miRNA-r in BC luminal A

Figure 4 shows the inter-pathway coordination among the final top 10 pairwise pathways in luminal A. Among pathways, the role of Glioma Invasiveness Signalling, hub of a network linking Tryptophan Degradation X (Mammalian, via Tryptamine), Ethanol Degradation IV, Dopamine Deregulation, Oxidative Ethanol Degradation III, and Fatty Acid-oxidation appears dominant.

We found six pairwise pathways with 11 significant miRNA regulators: 1) Acute Phase Response Signalling; HIF1 Signalling, 2) Axonal Guidance Signalling; Acute Phase Response Signalling 3) Ethanol Degradation IV; Glioma Invasiveness Signalling, 4) Ethanol Degradation IV; Estrogen Receptor Signalling 5) Glioma Invasiveness Signalling; Oxidative Ethanol Degradation III, and 6) Extrinsic Prothrombin Activation; Intrinsic Prothrombin Activation. Four pairwise pathways were not significantly deregulated by any miRNA.

Table 2 lists, for each of the six above mentioned pairwise pathways, their miRNA-r regulators with, their expression levels in BC luminal A and in NS, and the statistical significance of the comparison (in terms of log Fold Change).

**Table 2:** For each top 6 pairwise pathway in luminal A: miRNA regulators of pathways, their expression levels in BC and in NS, and the statistical significance of the comparison (in terms of log Fold Change)

Pairwise pathways	miRNA-r	miRNA-r Exp. in BC	miRNA-r Exp. in NS	Statistical significance (log Fold Change)
1. a) Acute Phase Response Signalling; b) HIF1 Signalling	*Hsa-miR-205*	13006.16	25001.9	-1.01017
2. a) Axonal Guidance Signalling; b) Acute Phase Response Signalling	*Hsa-miR-452* *Hsa-miR-335* *Hsa-miR-205* *Hsa-miR-99a* *Hsa-miR-337* *Hsa-miR-1250*	101.1974 333.4506 13006.16 4268.871 208.6352 0.746781	720.023 1391.644 25001.9 10953.55 547.3908 0.103448	-3.08782 -2.27528 -1.01017 -1.71181 -1.57547 1.636118
3. a) Ethanol Degradation IV; b) Glioma Invasiveness Signalling	*Hsa-miR-3199-1*	1.793991	5.45977	-1.79743
4. a) Ethanol Degradation IV; b) Estrogen Receptor Signalling	*Hsa-miR-1-1*	0.021459	1.62069	-3.82055
5. a) Glioma Invasiveness Signalling; b) Oxidative Ethanol Degradation III	*Hsa-miR-3199-1*	1.793991	5.45977	-1.79743
6. a) Extrinsic Prothrombin Activation; b) Intrinsic Prothrombin Activation	*Hsa-miR-1537* *Hsa-miR-210* *Hsa-miR-99a* *Hsa-miR-381*	0.957082 1592.884 4268.871 138.0215	0.333333 317.8046 10953.55 259.954	1.091521 2.397213 -1.71181 -1.25098

The results of the MI analysis for the identification of candidate genes target of miRNAs are shown in the Additional file 1.

### Luminal B vs. Normal

After 50 bootstraps, we found 129 pairwise pathways enriched with 5590 differentially expressed genes. Similarly to Luminal A. Many pathways were found in common among the top 10 pairs in many bootstraps.

The final top 10 pairwise pathways selected according to their frequency in the top 10 in all bootstraps from the 129 pairwise pathways, are shown in Table 3.

**Table 3:** Luminal B: frequency of pairwise pathways in the top 10 for all 50 bootstraps

Pairwise pathway	Frequency
1) Wnt/catenin Signalling;Mitotic Roles of Polo-Like Kinase	46/50
2) Epithelial Adherens Junction Signalling; Mitotic Roles of Polo-Like Kinase	39/50
3) Mitotic Roles of Polo-Like Kinase; Growth Hormone Signalling	26/50
4) Wnt/catenin Signalling;Cell Cycle Control of Chromosomal Replication	20/50
5) LPS/IL1 Mediated Inhibition of RXR Function; Assembly of RNA Polymerase II Complex	11/50
6) Calcium Signalling;Cell Cycle Control of Chromosomal Replication	11/50
7) Mitotic Roles of Polo-Like Kinase;RhoA Signalling	11/50
8) Epithelial Adherens Junction Signalling; Cell Cycle Control of Chromosomal Replication	10/50
9) Mitotic Roles of Polo Like Kinase; Factor Promoting Cardiogenesis in Vertebrates	9/50
10) Epithelial Adherens Junction Signalling; EIF2 Signalling	9/50
11) Acute Phase Response Signalling; HIF1 Signalling	9/50
12) Cellular Effects of Sildenafil (Viagra); Cell Cycle Control of Chromosomal Replication	9/50
13) ILK Signalling;Mitotic Roles of Polo-Like Kinase	9/50
14) Glioblastoma Multiforme Signalling; Mitotic Roles of Polo-Like Kinase	8/50
15) LPS/IL-1 Mediated Inhibition of RXR Function; linolenate Biosynthesis II (Animals)	7/50
….	
50)…	1/50

Dots indicate the other pairs of pathways with minor frequency

**Fig. 5:**
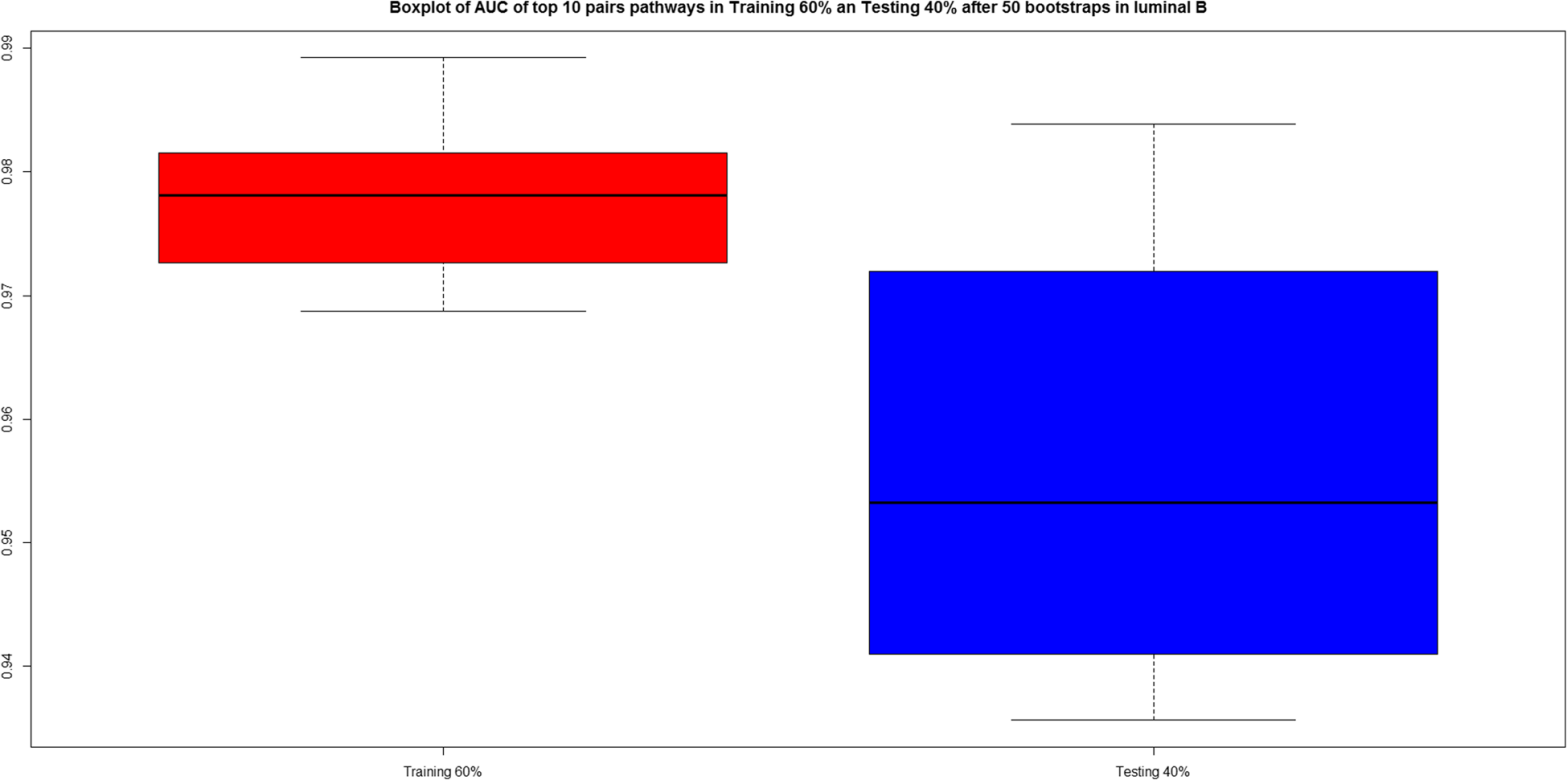
Boxplot of AUC values for the top 10 enriched pairwise pathways in luminal B, after all 50 bootstraps

Figure 5 shows a boxplot with the AUC values for the final 10 pairwise pathways in both the training and testing phase, confirming the good AUC (median >95 %) both for training and testing.

**Fig. 6:**
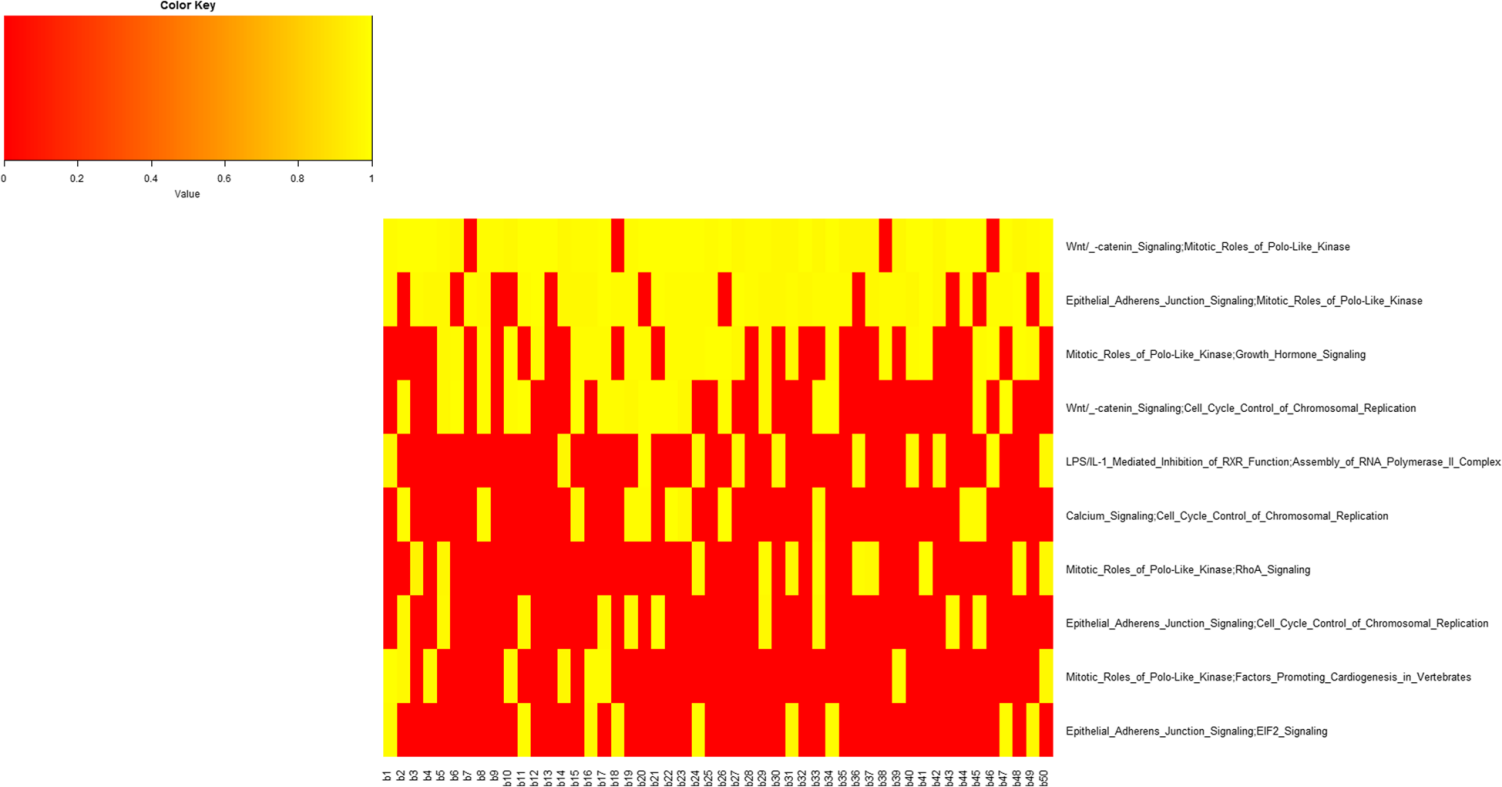
AUC values representation with the top 10 pairwise pathways for all 50 bootstraps in luminal B. *Yellow square* indicates AUC values when the pairwise pathway was included in the top 10 for the corresponding. *Red square* indicates that the pairwise pathway was not present in top 10 for that bootstrap

Figure 6 shows, for each bootstrap, the AUC of the top 10 pairs of pathways. We can see that some pairwise pathways (e.g. Wnt/ -catenin Signalling; Mitotic Roles of Polo-Like Kinase and Epithelial Adherens Junction Signalling; Mitotic Roles of Polo-Like Kinase) have excellent AUC in most bootstraps.

**Fig. 7:**
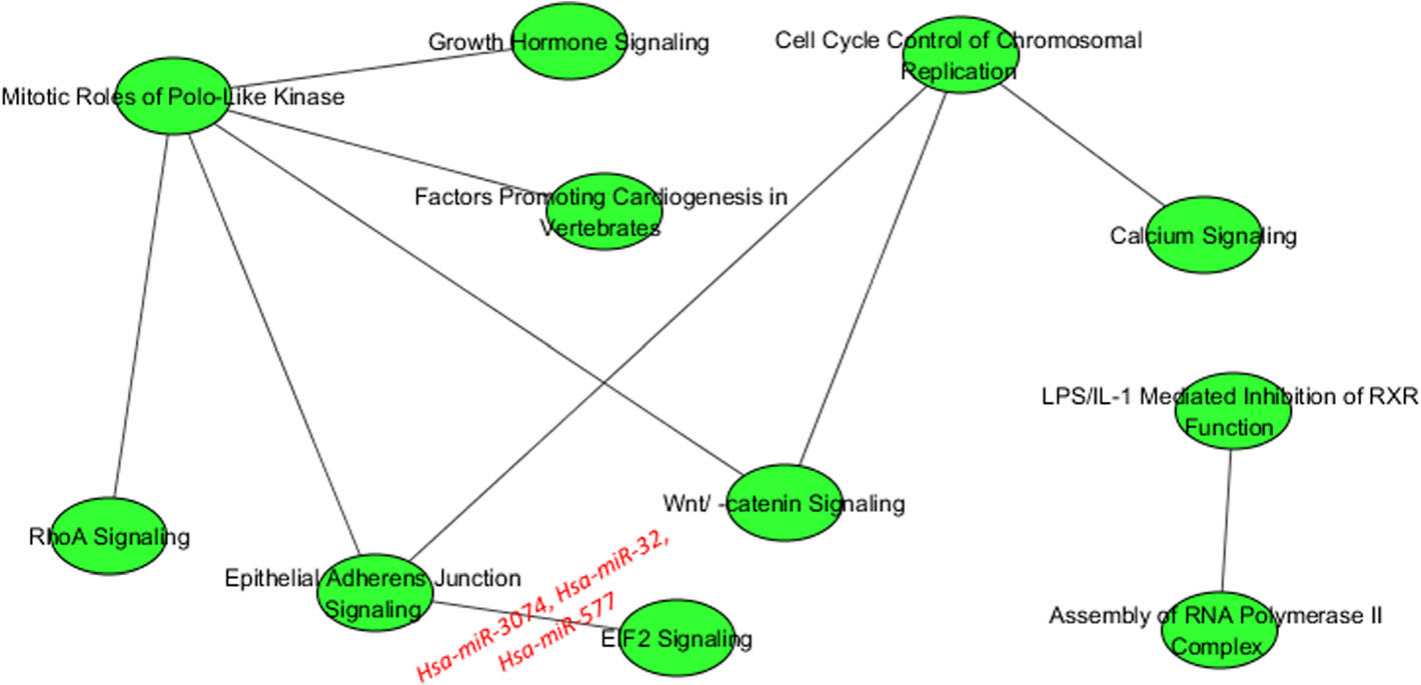
Interaction of the top 10 pairwise pathways in luminal B and their miRNA-r in luminal B BC

Figure 7 shows the inter-pathway coordination among the top 10 pairwise pathways in luminal B. We found only 3 miRNAs significantly deregulating 1 pairwise pathway (Epithelial Adherens Junction Signalling; EIF2 Signalling) which are also shown. Among pathways, the role of Mitotic Roles of Polo-like Kinase, hub of a network linking RhoA Signalling, Epithelial Adherens Junction Signalling, Wnt/catenin Signalling, Factors Promoting Cardiogenesis in Vertebrates, and Growth Hormone Signalling appears dominant.

Table 4 lists, for the above mentioned pairwise pathway, its miRNA regulators, their expression levels in BC luminal B and in NS, and the statistical significance of the comparison (in terms of log Fold Change).

**Table 4:** For the top pairwise pathway in luminal B: miRNA regulators of the pathways, their expression levels in BC and in NS, and the statistical significance of the comparison (in terms of log Fold Change)

Pairwise pathways	miRNA-r	miRNA-r Exp. in BC	miRNA-r Exp. in NS	Statistical significance (log Fold Change)
1. a) Epithelial Adherens Junction Signalling; b) EIF2 Signalling	*Hsa-miR-32* *Hsa-miR-3074* *Hsa-miR-577*	114.3883 77.45631 4.640777	50.54023 33.32184 15.78161	1.579455 1.368266 -1.48828

The results of the MI analysis for the identification of candidate genes target of miRNAs are shown in the Additional file 2.

### Basal vs. Normal

After 50 bootstraps, we found 74 pairwise pathways enriched with 6011 differentially expressed genes, since many pathways were found in common among the top 10 pairs in many bootstraps.

The final top 10 pairwise pathways, selected according to their frequency in the top 10 in all bootstraps from the 74 pairwise pathways, are shown in Table 5.

**Table 5:** Basal: frequency of pairwise pathways in the top 10 for all 50 bootstraps

Pairwise pathway	Frequency
1) Ethanol Degradation IV; Role of BRCA1 in DNA Damage Response	41/50
2) Putrescine Degradation III; Mismatch Repair in Eukaryotes	40/50
3) Ethanol Degradation IV; Mismatch Repair in Eukaryotes	36/50
4) Role of BRCA1 in DNA Damage Response; Oxidative Ethanol Degradation III	35/50
5) Ethanol Degradation II; Role of BRCA1 in DNA Damage Response	31/50
6) Role of BRCA1 in DNA Damage Response; Histamine Degradation	24/50
7) Tryptophan Degradation X (Mammalian, via Tryptamine); Role of BRCA1 in DNA Damage Response	24/50
8) Putrescine Degradation III;Role of BRCA1 in DNA Damage Response	23/50
9) Role of BRCA1 in DNA Damage Response; Putrescine Degradation III	18/50
10) Cell Cycle Control of Chromosomal Replication; Cellular Effects of Sildenafil (Viagra)	17/50
11) Cell Cycle Control of Chromosomal Replication; Colorectal Cancer Metastasis Signalling	16/50
12) Oxidative Ethanol Degradation III; Role of BRCA1 in DNA Damage Response	14/50
13) Role of BRCA1 in DNA Damage Response; Ethanol Degradation II	13/50
14) Cell Cycle Control of Chromosomal Replication; eNOS Signalling	12/50
15) Mismatch Repair in Eukaryotes; Fatty Acid -oxidation I	12/50
....	
50)....	1/50

Dots indicate the other pairs of pathways with minor frequency

**Fig. 8:**
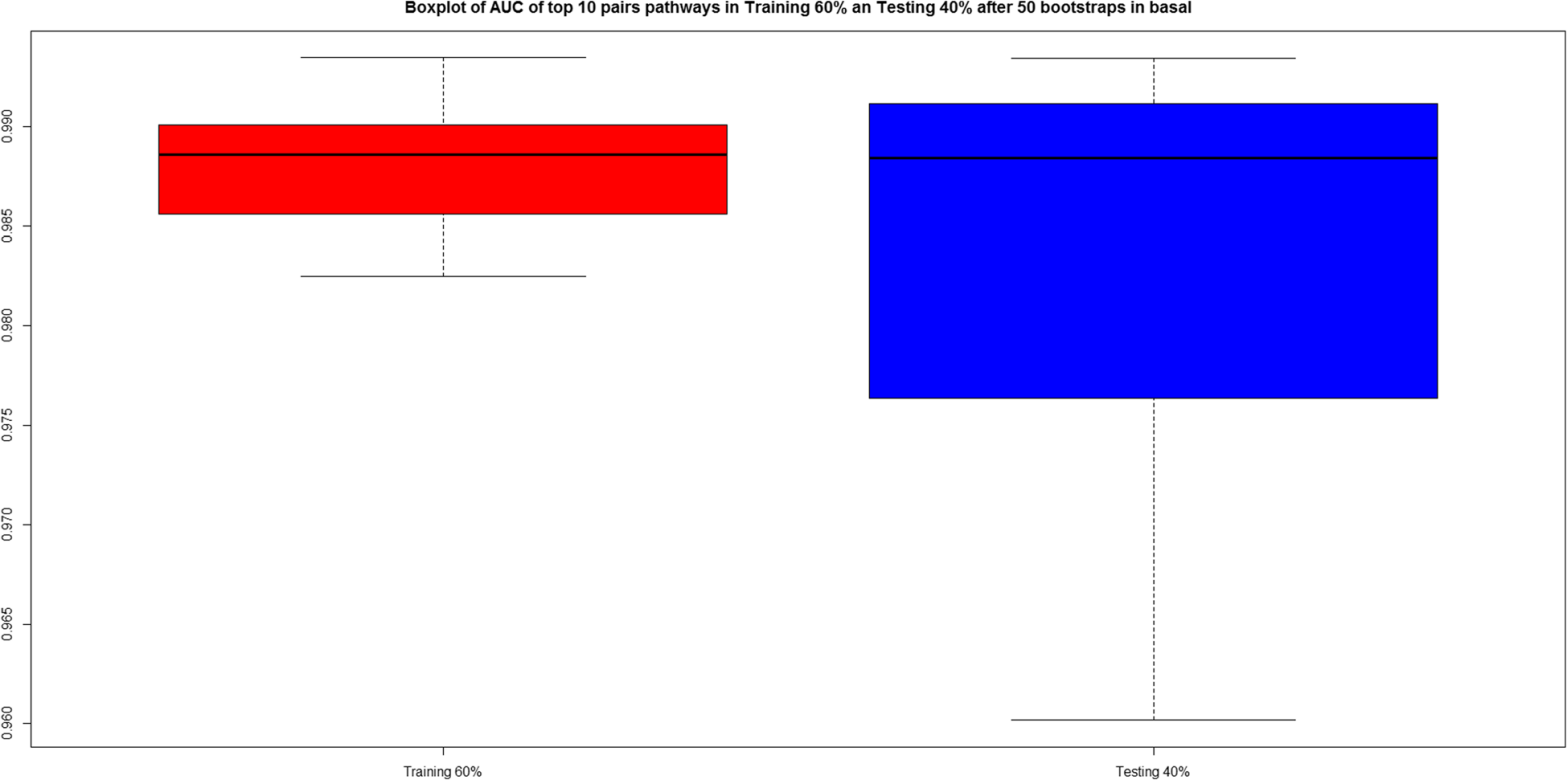
Boxplot of AUC values for the top 10 enriched pairwise pathways in basal, after all 50 bootstraps

Figure 8 shows a boxplot with the AUC values for the final 10 pairwise pathways in both the training and testing phase, confirming the good AUC (median >95 %) both for training and testing.

**Fig. 9:**
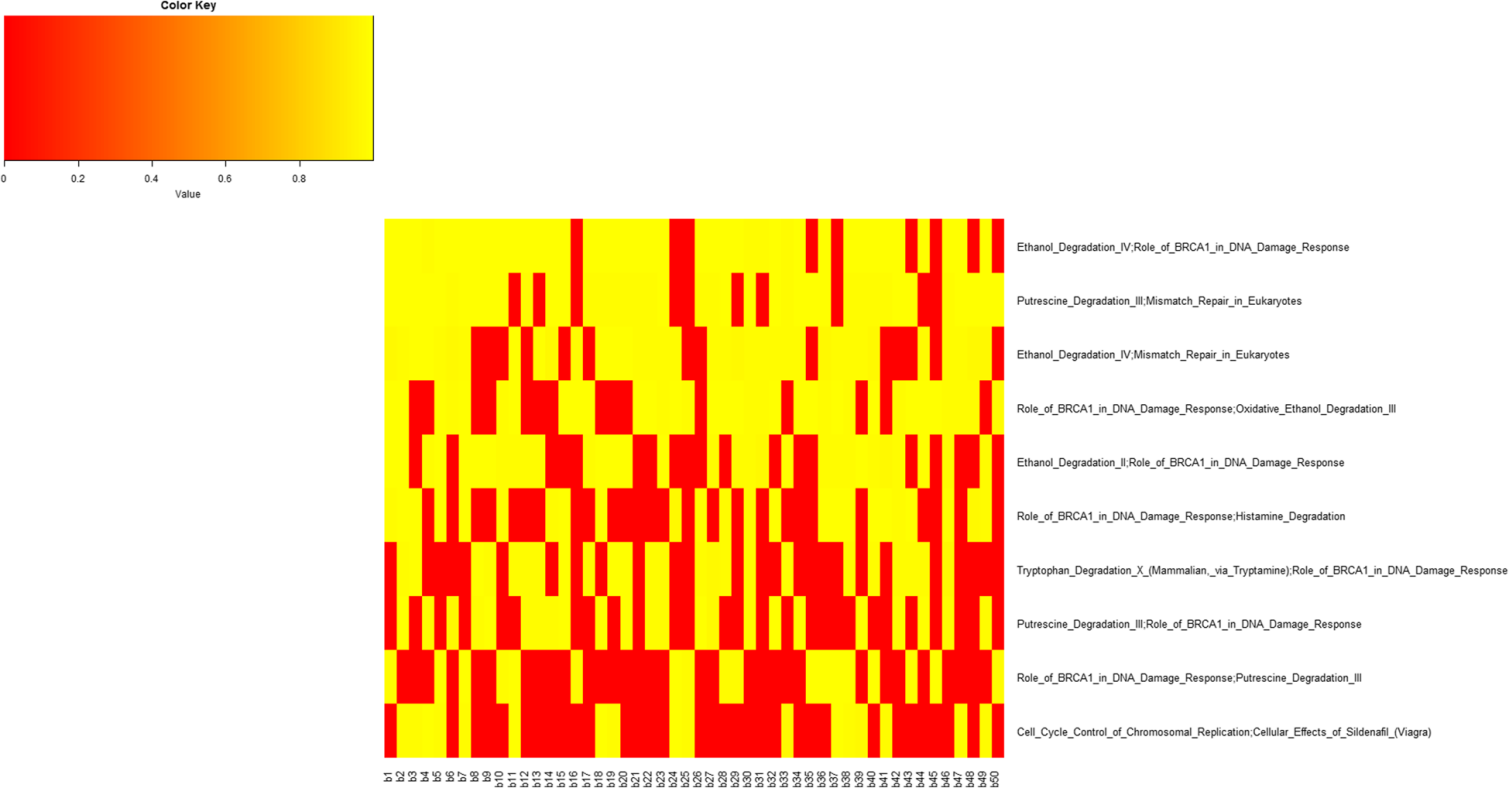
AUC values representation with the top 10 pairwise pathways for all 50 bootstraps in basal. *Yellow square* indicates AUC values when the pairwise pathway was included in the top 10 for the corresponding bootstrap. *Red square* indicates that the pairwise pathway was not present in top 10 for that bootstrap

Figure 9 shows, for each bootstrap, the AUC of the top 10 pairs of pathways, confirming also for basal, that some pairwise pathways (e.g. Ethanol Degradation IV; Role of BRCA1 in DNA Damage Response, and Putrescine Degradation III; Mismatch Repair in Eukaryotes) have excellent AUC in most bootstraps.

**Fig. 10:**
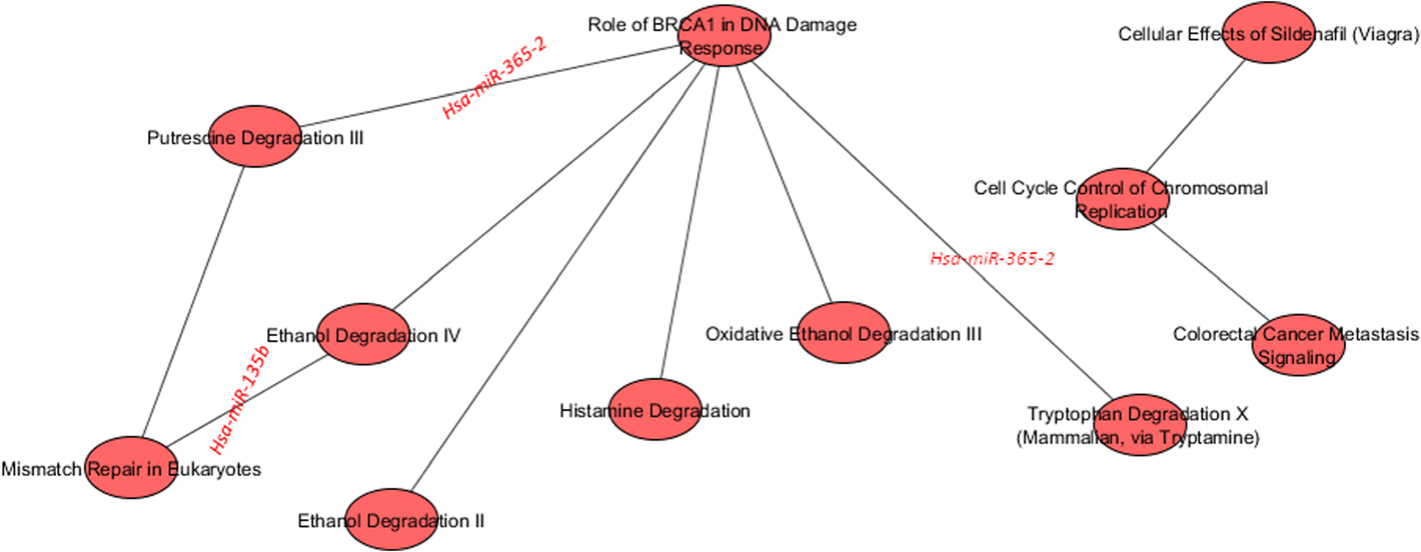
Interaction of the top 10 pairwise pathways in BC basal and their miRNA-r in BC basal

Figure 10 shows the inter-pathway coordination among the top 10 pairwise pathways in BC basal. We found only 2 miRNAs significantly deregulating 3 pairwise pathways: 1) Ethanol Degradation IV; Mismatch Repair in Eukaryotes, 2) Putrescine Degradation III; Role of BRCA1 in DNA Damage Response, 3) Tryptophan Degradation X (Mammalian, via Tryptamine); Role of BRCA1 in DNA Damage Response, which are also shown. Among pathways, the role of BRCA1 in DNA Damage Response, hub of a network linking Putrescine Degradation III, Ethanol Degradation IV, Ethanol Degradation II, Histamine Degradation, Oxidative Ethanol Degradation III, and Tryptophan Degradation X appears dominant.

Table 6 lists, for the 3 above mentioned pairwise pathways, miRNA regulators of pathways, their expression levels in BC basal and in NS, and the statistical significance of the comparison (in terms of log Fold Change).

**Table 6:** For each top 3 pairwise pathway in BC basal: miRNA regulators of pathways, their expression levels in BC and in NS, and the statistical significance of the comparison (in terms of log Fold Change)

Pairwise pathways	miRNA-r	miRNA-r Exp. in BC	miRNA-r Exp. in NS	Statistical significance (log Fold Change)
1. a) Ethanol Degradation IV; b) Mismatch Repair in Eukaryotes	*Hsa-miR-135b*	342.0541	13.01149	4.96603
2. a) Putrescine Degradation III; b) Role of BRCA1 in DNA Damage Response	*Hsa-miR-365-2*	153.5541	506.0575	-1.39949
3. a) Tryptophan Degradation X (Mammalian, via Tryptamine); b) Role of BRCA1 in DNA Damage Response	*Hsa-miR-365-2*	153.5541	506.0575	-1.39949

The results of the MI analysis for the identification of candidate genes target of miRNAs are shown in the Additional file 3.

### HER2 vs. Normal

After 50 bootstraps, we found 222 pairwise pathways enriched with 4464 differentially expressed genes.

The final top 10 pairwise pathways, selected according to their frequency in the top 10 in all bootstraps from the 222 pairwise pathways, are shown in Table 7.

**Table 7:** HER2: frequency of pairwise pathways in the top 10 for all 50 bootstraps

Pairwise pathway	Frequency
1) Axonal Guidance Signalling; CXCR4 Signalling	18/50
2) Atherosclerosis Signalling; Acute Phase Response Signalling	16/50
3) Role of Macrophages, Fibroblasts and Endothelial Cells in Rheumatoid Arthritis; Growth Hormone Signalling	12 /50
4) HIF1 Signalling; Glioblastoma Multiforme Signalling	11/50
5) Putrescine Degradation III; NAD biosynthesis II (from tryptophan)	11/50
6) HIF1 Signalling; Growth Hormone Signalling	11/50
7) Axonal Guidance Signalling; P2Y Purigenic Receptor Signalling Pathway	10/50
8) Acute Phase Response Signalling; HIF1 Signalling	8/50
9) Axonal Guidance Signalling; Growth Hormone Signalling	7/50
10) Cellular Effects of Sildenafil (Viagra); tRNA Charging	7/50
11) Hepatic Fibrosis/Hepatic Stellate Cell Activation; Coagulation System	6/50
12) Acute Phase Response Signalling; Role of Macrophages, Fibroblasts and Endothelial Cells in Rheumatoid Arthritis	6/50
13) Retinoate Biosynthesis I; Estrogen Receptor Signalling	6/50
14) Factors Promoting Cardiogenesis in Vertebrates; tRNA Charging	6/50
15) Role of Macrophages, Fibroblasts and Endothelial Cells in Rheumatoid Arthritis; Role of BRCA1 in DNA Damage Response	6/50
…	
50)....	1/50

Dots indicate the other pairs of pathways with minor frequency

**Fig. 11:**
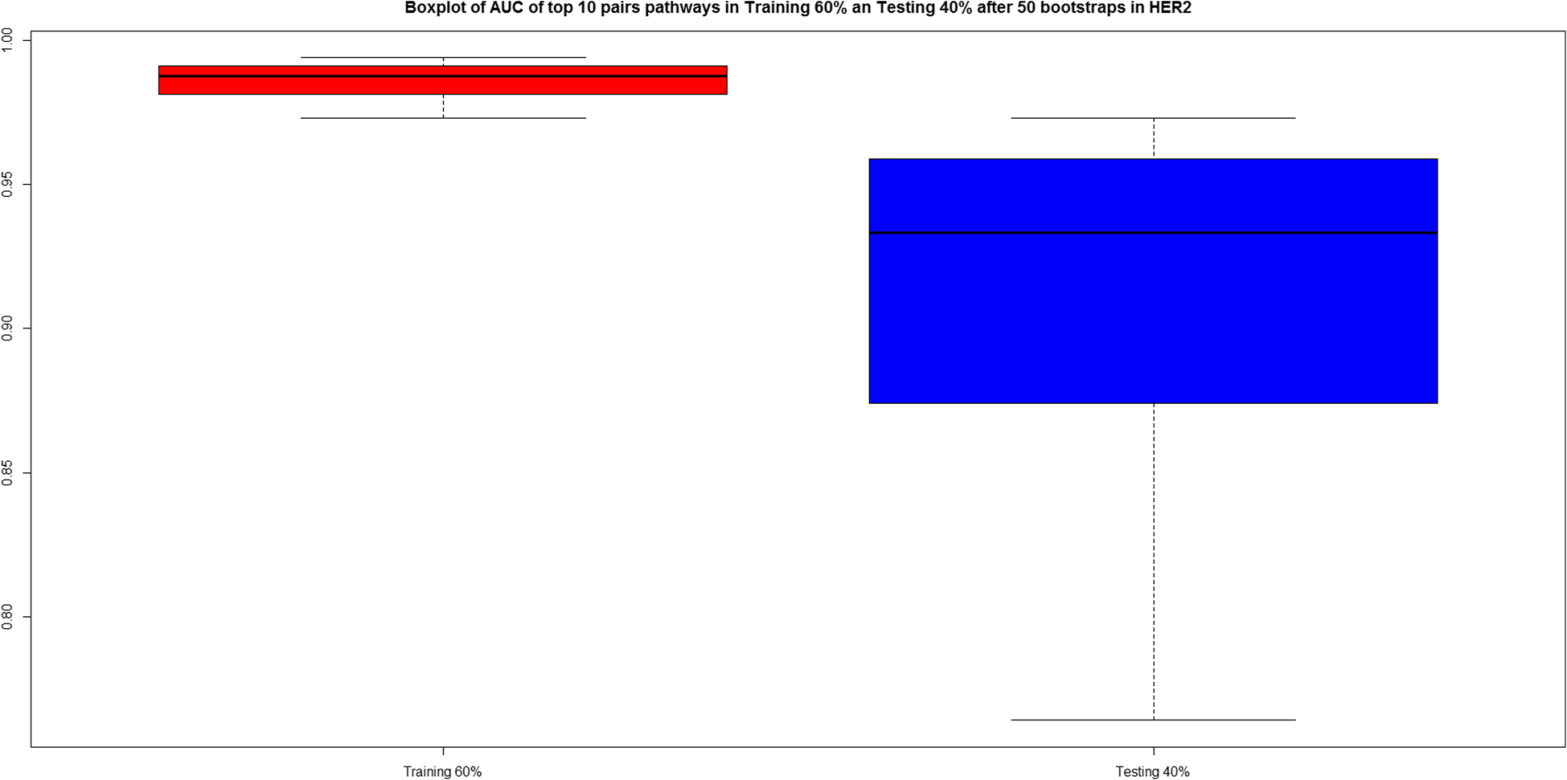
Boxplot of AUC values for the top 10 enriched pairwise pathways in HER2, after all 50 bootstraps

Figure 11 shows a boxplot with the AUC values for the final 10 pairwise pathways in both the training and testing phase, confirming good AUC (median >90 %), both for training and testing.

**Fig. 12:**
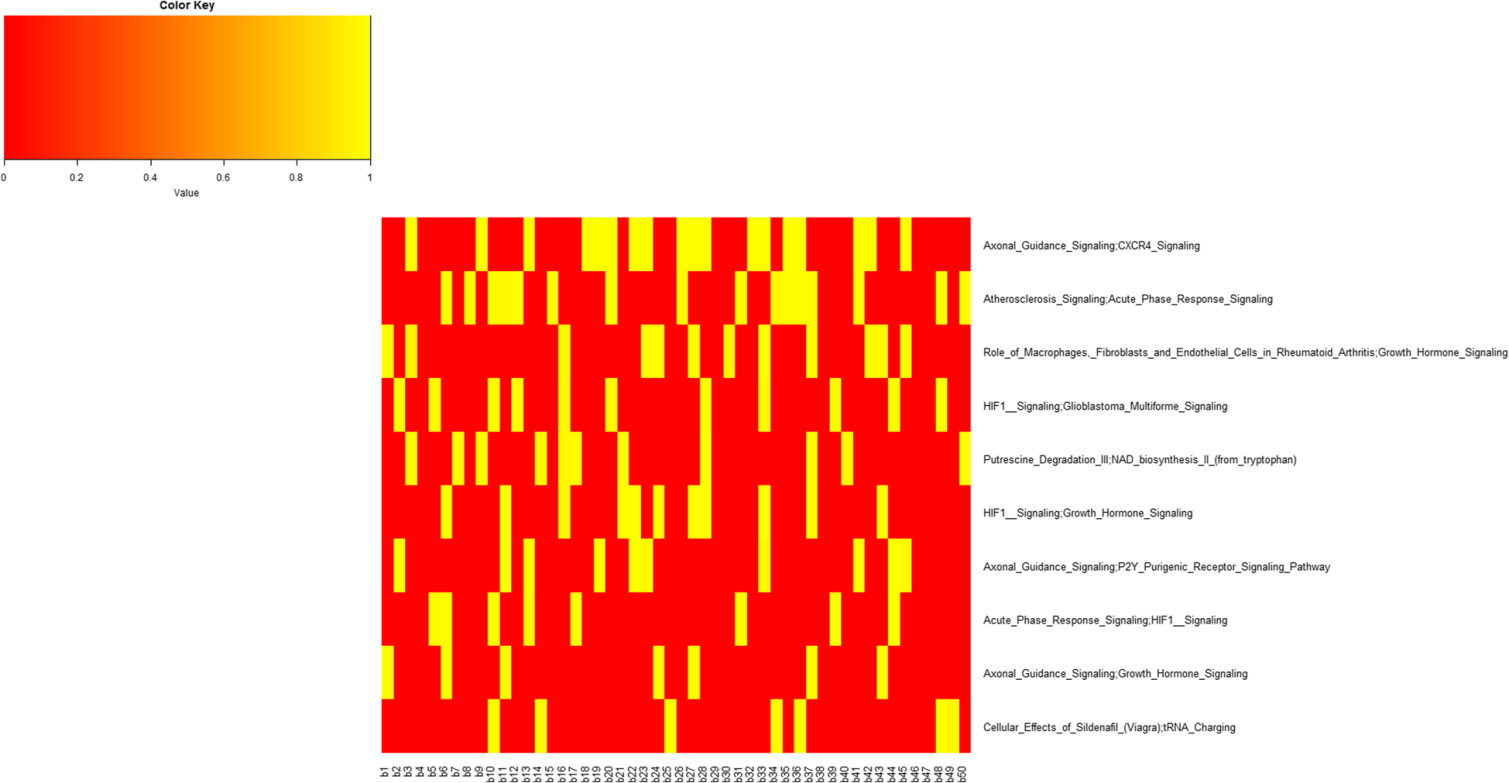
AUC values representation with the best top 10 pairwise pathways for all 50 bootstraps in HER2. *Yellow square* indicates AUC values when the pairwise pathway was included in the top 10 for the corresponding bootstrap. *Red square* indicates that the pairwise pathway was not present in top 10 for that bootstrap

Figure 12 shows, for each boostrap, the AUC of the top 10 pairs of pathways, confirming that some pairwise pathways (e.g. Axonal Guidance Signalling;CXCR4 Signalling, Atherosclerosis Signalling; Acute Phase Response Signalling) have excellent AUC in most bootstraps.

**Fig. 13:**
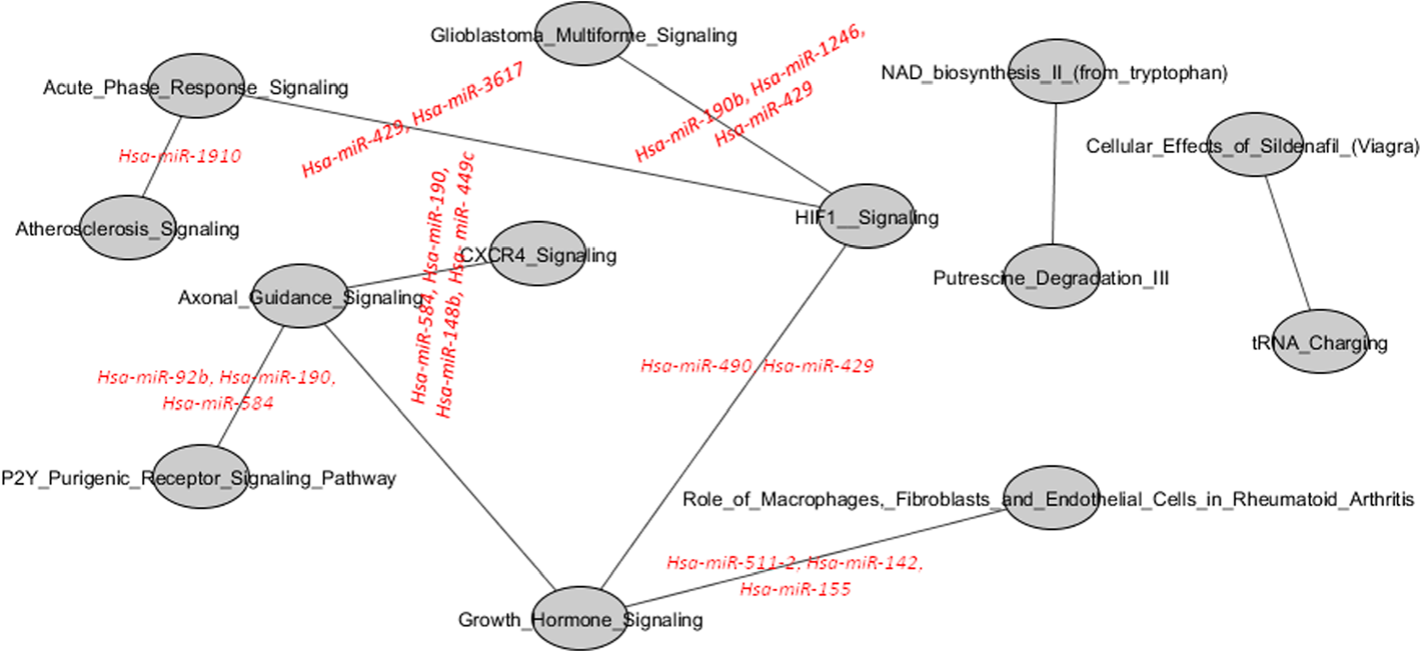
Interaction of the top 10 pairwise pathways in HER2 BC and their miRNA-r in BC HER2

Figure 13 shows the inter-pathway coordination among the top 10 pairwise pathways in BC HER2. We found 14 miRNAs significantly deregulating 7 pairwise pathways which are also shown 1) Acute Phase Response Signalling; HIF1 Signalling, 2) Atherosclerosis Signalling; Acute Phase Response Signalling, 3) Axonal Guidance Signalling; CXCR4 Signalling, 4) Axonal Guidance Signalling; P2Y Purigenic Receptor Signalling Pathway 5) HIF1 Signalling; Glioblastoma Multiforme Signalling 6) HIF1 Signalling; Growth Hormone Signalling, 7) Role of Macrophages, Fibroblasts and Endothelial Cells in Rheumatoid Arthritis; Growth Hormone Signalling. Among pathways, the role of Growth Hormone Signalling, hub of a network linking Axonal Guidance Signalling, HIF1 Signalling, Role of Macrophages, Fibroblasts and Endothelial Cells in Rheumatoid Arthritis appears dominant.

Table 8 lists, for each of the seven pairwise pathways, miRNA regulators of pathways, their expression levels in BC HER2 and in NS, and the statistical significance of the comparison (in terms of log Fold Change).

**Table 8:** For each top 7 pairwise pathway in BC HER2: miRNA regulators of pathways, their expression levels in BC and in NS, and the statistical significance of the comparison (in terms of log Fold Change)

Pairwise pathways	miRNA-r	miRNA-r Exp. in BC	miRNA-r Exp. in NS	logFC
1. a) Acute Phase Response Signalling; b) HIF1 Signalling	*Hsa-miR-429* *Hsa-miR-3617*	434.2326 0.209302	71.18391 0.045977	2.604998 1.078421
2. a) Atherosclerosis Signalling; b) Acute Phase Response Signalling	*Hsa-miR-1910*	1.581395	0.425287	1.501305
3. a) Axonal Guidance Signalling; b) CXCR4 Signalling	*Hsa-miR-584* *Hsa-miR-190* *Hsa-miR-148b* *Hsa-miR-449c*	152.5116 11.60465 719.4651 5.651163	482.4253 31.05747 315.1839 0.068966	-1.30985 -1.02572 1.550205 5.430282
4. a) Axonal Guidance Signalling; b) P2Y Purigenic Receptor Signalling Pathway	*Hsa-miR-92b* *Hsa-miR-190* *Hsa-miR-584*	531.8605 11.60465 152.5116	171.1494 31.05747 482.4253	1.840728 -1.02572 -1.30985
5. a) HIF1 Signalling; b) Glioblastoma Multiforme Signalling	*Hsa-miR-190b* *Hsa-miR-1246* *Hsa-miR-429*	31.90698 0.255814 434.2326	6.137931 0.034483 71.18391	2.680854 1.277809 2.604998
6. a) HIF1 Signalling; b) Growth Hormone Signalling	*Hsa-miR-490* *Hsa-miR-429*	5.395349 434.2326	0.298851 71.18391	4.276816 2.604998
7. a) Role of Macrophages. Fibroblasts and Endothelial Cells in Rheumatoid Arthritis; b) Growth Hormone Signalling	*Hsa-miR-511-2* *Hsa-miR-142* *Hsa-miR-155*	17.69767 11699.28 1931.163	56.51724 3246.46 616.9425	-1.49388 2.248187 1.707846

The results of the MI analysis for the identification of candidate genes target of miRNAs are shown in the Additional file 4.

## Discussion

In a normal condition the biological pathways act in a coordinated way to collaborate in a biological process. Cancer can interfere in these coordinated processes, since alterations in multiple genes that participate in different pathways result in an uncontrolled growth of tumour cells, invasion and metastases.

In this work, by assessing the coordination among different pathways deregulated in BC subtypes, we observed key pairwise pathways for each BC subtype, which enabled the identification of a network of dependent pathways characteristic of the disease. Furthermore, we identified miRNAs that control this network with a potential role in BC. These miRNAs could be crucial modulators of upstream signalling events linked by specific subtype of BC.

### miRNAs regulating pathways in luminal A

In BC luminal A we identified 11 miRNAs (*Hsa-miR-1-1, Hsa-miR-1250, Hsa-miR-1537, Hsa-miR-205, Hsa-miR-210, Hsa-miR-3199-1, Hsa-miR-335, Hsa-miR-337, Hsa-miR-381, Hsa-miR-452, and Hsa-miR-99a*) which could be key modulators of six pairs of pathways: 1) Intrinsic Prothrombin Activation, and Extrinsic Prothrombin Activation; 2) Acute Phase Response Signalling, and HIF1 Signalling 3) Axonal Guidance Signalling, and Acute Phase Response Signalling; 4) Ethanol Degradation IV, and Glioma Invasiveness Signalling; 5) Ethanol Degradation IV, and Estrogen Receptor Signalling; and 6) Glioma Invasiveness Signalling, and Oxidative Ethanol Degradation III.

#### Intrinsic prothrombin activation and extrinsic prothrombin activation, regulated by *Hsa-miR-99a*, *Hsa-miR-210*, *Hsa-miR-381*, and *Hsa-miR-1537*

Intrinsic and extrinsic prothrombin activation pathways perform an essential role in coagulation, an important process for the establishment of metastasis also in experimental models of cancer [70].

*Hsa-miR-99a* has been already associated with BC; in particular, its up regulation correlates with cells with stemness properties [71]. Moreover, *Hsa-miR-99a* has been identified in a screening of miRNA profiles able to discriminate ductal carcinoma in situ, invasive BC, metastatic BC and normal tissues. *Hsa-miR-99a* shows specific differential expression in the in situ subtype of BC [72]. Among the other miRNAs regulating the two above mentioned pathways, *Hsa-miR-381* is one of the possible circulating miRNAs able to discriminate between blood samples of patients with BC and NC [73]. *Hsa-miR-210* was demonstrated to be a potential predictor of the outcome of different cancers, including BC [74]. Circulating *Hsa-miR-210* levels were associated with trastuzumab sensitivity, tumour presence, and lymph node metastases, suggesting *Hsa-miR-210* as a predictor and perhaps a monitor of the response to therapies including trastuzumab [75].

Limited data about *Hsa-miR-1537* is available for this miRNA. Few publications, not related to BC, described this miRNA as altered in the serum and bile of cholangioma patients [76], where a speculation of a role for *Hsa-miR-1537* in inflammation is proposed.

#### Acute phase response signalling and HIF1 signalling, regulated by *Hsa-miR-205*

The acute phase response is a rapid inflammatory response that provides protection against infections, including cancer [77]. Several studies found cross-talk between HIF-1 signalling and inflammatory pathways suggesting that the development of inflammation in response to hypoxia is clinically relevant [78].

*Hsa-miR-205* is an oncosuppressive miRNA lost in BC; it is directly transactivated by the oncosuppressor p53 [79]. Sempere et al. [80] showed that *Hsa-miR-205* expression is restricted to the myoepithelial/basal cell compartment of normal mammary ducts and lobules, and is reduced or completely eliminated in matching tumour specimens. *Hsa-miR-205* regulates a number of important oncogenic targets as *ZEB1*, *VEGFA*, and *HER3*. Moreover, it may modulate additional targets, such as HMGB3, showing a potential therapeutic benefit/role [81].

#### Axonal guidance signalling and acute phase response signalling, regulated by *Hsa-miR-205*, *Hsa-miR-99a*, *Hsa-miR-335*, *Hsa-miR-337*, *Hsa-miR-452*, and *Hsa-miR-1250*

We found that two pathways, Axonal Guidance Signalling and Acute Phase Response Signalling, are regulated by a group of 6 miRNAs.

There are four families of secreted or membrane-bound factors with repulsive or attractive activities for growing axons and migrating neurons (i.e., netrin 1, semaphorine, ephrins, and Slit, all with their receptors), which have recently emerged as pivotal factors in tumour progression. Far from being confined to the developing brain, axonal guidance signalling seems to play an important role in tumour cell migration, tumour cell survival and tumour angiogenesis [82].

*Hsa-miR-205* has already been proposed as a circulating biomarker of the response of the BC luminal A subtype to neoadjuvant chemotherapy [83].

*Hsa-miR-335* is down regulated in cancer stem cells (CSC) targeting genes, such as *Bmi1* and *Suz12* component, *Zeb1/2*, and *Klf4*, all belonging to a regulatory circuit that sustains the breast CSC state [84]. *Hsa-miR-335* could be used as prognostic marker [85] and could suppress neuroblastoma cell invasiveness by directly targeting multiple genes from the non-canonical TGF- β signalling pathway [86].

*Hsa-miR-337* plays a role in the reduction of gastric cancer cell invasion capacity and its loss has been associated with lymph node metastasis [87]. Furthermore, a study in prostate cancer revealed *Hsa-miR-337* as a potential circulating biomarker able to identify risk groups [88].

*Hsa-miR-452* has been found to be associated with adriamycin-resistance of BC cells, at least, partially, by targeting the insulin-like growth factor-1 receptor (IGF-1R) [89], and contributes to the docetaxel resistance of BC cells [90].

*Hsa-miR-1250* has been described in the white matter tracts of the human brain. Although no publication is available regarding its role in BC, *Hsa-miR-1250* seems to perform a role in oligodendrocyte proliferation and differentiation [91].

#### Ethanol degradation IV, and glioma invasiveness signalling regulated by *Hsa-miR-3199-1*

Looking to the list of genes in Ethanol Degradation IV, we found that a lot of these genes belong to the family of *ALDH* genes, and, although as they perform a role in ethanol detoxification, they are also considered biomarkers of CSCs [92].

ER+ cells are able to generate cell progeny of luminal lineage both in vitro and in vivo. Loss of *ALDH* isoform, *ALDH1A1*, plays a role in this process by weakening cellular differentiation [93]. Several studies demonstrated that *ALDH1A1* correlates with ER status in BC, and that *ALDH1A1* is an independent predictor of poor clinical outcome [94, 95].

Looking at the list of genes involved in glioma invasiveness signalling, we found several genes such as *ITGB5*, belonging to the integrins family, the integrin signalling members (Rhoh, Rhou and VTN) or some members of phosphatidylinositol 3-kinase (*PI3K*) signalling (*PIK3C2B*, *PIK3CB*). Integrins comprise a large family of cell surface receptors and control cell attachment to the extracellular matrix (*ECM*), growth, differentiation, apoptosis, cell motility, migration and survival. A role for integrins in BC development has been already described [96]. *Rhoh* and *Rhou* proteins have a critical role in the tumour progression and invasion, being important for the transduction of the signal from integrins to the neighbourhood cell during cell-cell communication [97, 98]. The *PI3K* signalling pathway in BC is associated with the poor outcome luminal B subtype, as its activation leads to the development of endocrine therapy resistance [99].

As regards *Hsa-miR-3199-1*, no publication is available on BC or on other cancer types.

#### Ethanol degradation IV and estrogen receptor signalling, regulated by *Hsa-miR-1-1*

We have already previously discussed the role of genes comprised in the list of Ethanol Degradation IV pathway.

As regards the pathway of Estrogen Receptor Signalling, ERs are critical regulators of breast epithelial cell proliferation, differentiation, and apoptosis. Nowadays the role of ER pathway in BC malignancy development is quite clear. This is the reason why several therapeutic approaches have been directed against ER+ BC [100].

*Hsa-miR-1-1* has been demonstrated to be a tumour suppressor gene that represses cancer cell proliferation and metastasis and promotes apoptosis by ectopic expression [101]. *Hsa-miR-1-1* regulates downstream functions of oncogenic signalling pathways such as Met, HDAC4, PIM-1, Wnt, Cyclin D, FOXP1, Slug, and TAGLN2 [101]. Down regulation of *Hsa-miR-1-1* was found to be associated with colorectal cancer progression [102].

#### Glioma Invasiveness Signalling, and Oxidative Ethanol Degradation III regulated by *Hsa-miR-3199-1*

As regards the pathway of Glioma Invasiveness Signalling, we have already discussed the role of its genes previously.

Looking at the list of genes involved in Oxidative Ethanol Degradation III, regulated by *Hsa-miR-3199-1*, we found some isoforms of the Phosphatidylinositol 3-kinase (PI3K) protein. PI3K includes two subunits, *p85α* and *p110α*, that are mediators of the pro-survival PI3K/Akt pathway signalling. Some isoforms of *PI3K* as well as *p85* subunit have been already described in HER2-positive BC patients, responding to trastuzumab treatment [103]. Among the other genes, particularly interesting if the finding of ITGB5, an integrin belonging to a family of six genes (*ITGA3*, *ITGA6*, *ITGAv*, *ITGB3*, *ITGB4* and *ITGB5*), which control cell attachment to the extracellular matrix and play an important role in mediating cell proliferation, migration and survival [104]. A strong association between integrin expression, mutation or polymorphism and BC onset has been already described [104].

The two described pathways seem to be controlled by a common miR, *Hsa-miR-3199-1.* No publication is currently available about the function of this miRNA in any biological processes.

### miRNAs regulating pathway in luminal B

In BC luminal B, we identified 3 miRNAs (*Hsa-miR-32*, *Hsa-miR-3074*, and *Hsa-miR-577*), which could be key modulators of the pair of pathways Epithelial Adherens Junction Signalling - EIF2 Signalling.

#### Epithelial adherens junction signalling and EIF2 signalling regulated by *Hsa-miR-32*, *Hsa-miR-3074*, and *Hsa-miR-577*

Adherens junctions are specialist structures for cell-cell adhesion machinery. The adhesive process is directly related to the differentiation and normal development of the tissue [105]. The development of cancer represents a modification of normal tissue homeostasis and a change in cell-cell interaction. In addition, cancer metastasis spreads through the circulatory system due to cell adhesion [105].

EIF2 Signalling is an essential factor for translation initiation and protein synthesis. No study showed a correlation between these pathways.

*Hsa-miR-32* is located in genomic regions, which might be involved in malignancies via deletion, amplification, or epigenetic modification mechanisms [106]. It regulates phosphatase and tensin homologue (*PTEN*) expression, and promotes proliferation, migration and invasion in colorectal cancer [107].

*Hsa-miR-3074* has been associated with papillary renal cell carcinoma [108], but no publication is available about its role in BC.

*Hsa-miR-577* is mainly involved in proliferation control in glioblastoma [109], hepatocellular carcinoma [110] and in esophageal squamous cell carcinoma [111]. It is possible to hypothesise a role for *Hsa-miR-577* also in BC proliferation control.

### miRNAs regulating pathway in basal

In BC basal, we identified 2 miRNAs (*Hsa-miR-135b*, and *Hsa-miR-365-2*) that may play an important role in the regulation of three pairs of pathways: 1) Ethanol Degradation IV, and Mismatch Repair in Eukaryotes; 2) Putrescine Degradation III, and Role of BRCA1 in DNA Damage Response, and 3) Tryptophan Degradation X (Mammalian, via Tryptamine), and Role of BRCA1 in DNA Damage Response.

#### Ethanol degradation IV and mismatch repair in eukaryotes, regulated by *Hsa-miR-135b*

Mismatch Repair plays a key role in maintaining genomic stability. Cells possess multiple mechanisms to repair DNA damage and thus prevent mutations [112]. No study revealed a direct interaction between Ethanol Degradation IV, and Mismatch Repair in BC.

*Hsa-miR-135b* levels are elevated in a variety of cancers including BC [113]. Lowery et al. [113] identified a 15-miRNA predictive signature related to the expression of ER comprising also this miRNA (*Hsa-miR-135b*, *Hsa-miR-190*, *Hsa-miR-217*, *Hsa-miR-218*, *Hsa-miR-299*, and *Hsa-miR-342*). Up regulation of *Hsa-miR-135b* is more robust in highly invasive than less invasive lines. In colorectal cancer, *Hsa-miR-135b* promotes cancer progression by acting as a downstream effector of oncogenic pathways [114].

#### Putrescine degradation III, and role of BRCA1 in DNA damage response regulated by *Hsa-miR-365-2*

Putrescine is a known metabolite that plays an important role in cancer and CSCs [115]. Putrescine belongs to the class of polyamine, involved in numerous processes in normal and cancer cells, such as proliferation, apoptosis, cell-cell interactions, and angiogenesis [116]. An association between the basal subtype and BRCA1 gene has been well described, and it may suggest that both inherent DNA damage–sensing processes and DNA repair mechanisms are crucial in the development of basal-like tumours [22, 23].

*Hsa-miR-365-2* negatively regulates BCL2 protein levels, and its overexpression combined with the deregulation of other 2 miRNAs have an apoptotic effect thus suggesting a therapeutic potential [117]. In pancreatic cancer *Hsa-miR-365* was found to induce gemcitabine resistance by targeting the adaptor protein SHC1 and pro-apoptotic regulator BAX [118].

#### Tryptophan degradation X (Mammalian, via Tryptamine), and role of BRCA1 in DNA damage response regulated by *Hsa-miR-365-2*

Altered tryptophan metabolism is linked to cancer development and progression [119, 120]. In particular, indoleamine 2,3-dioxygenase 1 (IDO1), an enzyme involved in tryptophan degradation, has been documented to have therapeutic potential, alone or in combination with chemotherapy or immunotherapy [121]. Several studies confirmed its immunosuppressive role and the inhibition of the IDO1 pathway therefore represents a promising therapeutic approach. Clinical trials evaluating the first IDO1 inhibitors have already started [122, 123]. The role of *Hsa-miR-365* in cancer has been already reported above.

### miRNAs regulating pathway in HER2

In HER2 BC, we identified 14 miRNAs (*Hsa-miR-1246*, *Hsa-miR-142*, *Hsa-miR-148b*, *Hsa-miR-155*, *Hsa-miR-190*, *Hsa-miR-190b*, *Hsa-miR-1910*, *Hsa-miR-3617*, *Hsa-miR-429*, *Hsa-miR-449c*, *Hsa-miR-490*, *Hsa-miR-511-2*, *Hsa-miR-584*, *Hsa-miR-92b*) that may have an important role in the regulation of seven pairs of pathways: 1) Axonal Guidance Signalling; CXCR4 Signalling; 2) Axonal Guidance Signalling; P2Y Purigenic Receptor Signalling, 3) Role of Macrophages, Fibroblasts and Endothelial Cells in Rheumatoid Arthritis; Growth Hormone Signalling, 4) HIF1 Signalling; Growth Hormone Signalling, 5) HIF1 Signalling; Glioblastoma Multiforme Signalling, 6) Acute Phase Response Signalling; HIF1 Signalling, and 7) Atherosclerosis Signalling; Acute Phase Response Signalling.

#### Axonal guidance signalling and CXCR4 signalling, regulated by *Hsa-miR-148b*, *Hsa-miR-190*, *Hsa-miR-449c*, *Hsa-miR-584*

The role of Axonal Guidance Signalling in cancer has already been mentioned above. CXCR4 Signalling shows a down regulation in metastasised BC cells [124]. *CXCR4*, the receptor for stromal-derived factor-1, is already reported as involved in breast carcinogenesis and invasion. Recent studies showed that the inhibition of CXCR4 expression resulted in an anti-invasive effect revealing the potential for the treatment of BC [125].

*CXCR4*, the receptor of *SDF-1*, plays a crucial role in modulating axonal responsiveness through a cyclic nucleotide-dependent signalling pathway [126].

Four miRNAs could be important regulator of this interaction.

*Hsa-miR-148b* was found to be a major coordinator of malignancy influencing invasion, survival to anoikis, extravasation, lung metastasis formation, and chemotherapy response [127]. Circulating *Hsa-miR-148b* was validated and found elevated in the plasma of BC patients compared to healthy women [128, 129]. Cimino et al. [127] showed that *Hsa-miR-148b* expression enhances chemotherapy-induced apoptosis.

*Hsa-miR-190* was associated with lymph node metastasis and its increased expression inhibited cell migration and invasiveness. The target of *Hsa-miR-190* was protease-activated-receptor 1 (PAR-1), which is a metastasis promoting protein in several cancers [130].

*Hsa-miR-449c* showed a decreased expression in human gastric tumours and induces senescence and apoptosis by activating the p53 pathway [131]. No information is reported about *Hsa-miR-449c* and BC.

*Hsa-miR-584* was found to be down-regulating TGF-β in BC cells. PHACTR1, an actin-binding protein, is also regulated by *Hsa-miR-584*. Overexpression of *Hsa-miR-584* and knockdown of PHACTR1 resulted in a drastic rearrangement of the actin cytoskeleton and in a loss of TGF-β-induced cell migration [132].

#### Axonal guidance signalling and P2Y purigenic receptor signalling pathway, regulated by *Hsa-miR-190*, *Hsa-miR-584*, and *Hsa-miR-92b*

The role of Axonal Guidance Signalling in cancer has already been mentioned above. A recent study showed that P2Y Purigenic Receptor Signalling Pathway is included in a potential pathway signature for testing Gemcitabine (Gem)-based chemotherapies sensitivity of gallbladder cancer patients [133].

P2Y receptors (e.g., P2Y1, P2Y2) have strong direct effects on the tumour by modulating cell growth. In vivo data support in vitro evidence that lowering the intratumour adenosine concentration and targeting the P2X7 receptor have a strong antitumour effect [134]. No study showed a direct interaction between Axonal Guidance Signalling and P2Y Purigenic Receptor Signalling.

*Hsa-miR-190* has already been discussed above. As regards *Hsa-miR-584,* the involvement of this miRNA in the pathway controlled by TGF-β [132] has been already described. In particular, TGF-β is able to decrease the expression of *Hsa-miR-584*. This in turn leads to the increase of protein phosphatase and actin regulator 1 (PHACTR1), a protein required for TGF-β-induced cell migration of breast cancer cells [132]. The drastic reorganization of the actin cytoskeleton is important in axonal guidance signalling, playing a role in tumour cell migration, tumour cell survival and tumour angiogenesis.

*Hsa-miR-92b*, regulating these pairwise pathways, was found over-expressed in brain primary tumours, suggesting a functional link between neuronal stem cells and brain tumourigenesis [135]. The involvement of this miRNA in radiation resistance was also found [136].

#### Role of macrophages, fibroblasts and endothelial cells in rheumatoid arthritis and growth hormone signalling, regulated by *Hsa-miR-142*, *Hsa-miR-155*, *Hsa-miR-511-2*

Tumours comprise proliferating tumour cells and stromal cells, including endothelial cells, inflammatory cells, and fibroblasts [137].

Macrophages play a crucial role in the innate and adaptive response to pathogens. Recently, it was also found that tumour-associated macrophages interact with CSCs thus leading to tumourigenesis, metastasis, and drug resistance [138]. As regards *Hsa-miR-511-2*, several publications demonstrated that this miRNA plays an important role in modulating tumour-associated macrophages. The upregulation of *Hsa-miR-511* affects the pro-tumoural gene signature of tumour-associated macrophages, which are endowed with tissue-remodelling, proangiogenic, and protumoural activity [139, 140].

Hormones play an important role for normal development and possibly also for tumour formation in the mammary gland. Human growth hormone could also stimulate the tumour initiating capacity and metastasis of estrogen receptor-negative BC [141]. *Hsa-miR-142* plays a role as potent inhibitor of human growth hormone signalling in normal and cancer cells thus suggesting the development of miRNA inhibitors as therapeutic agents in growth hormone-related disease, including cancer [142].

*Hsa-miR-155*, described as oncomiR, is implicated in EMT, cell migration, and invasion control. Roth et al. [143] found *Hsa-miR-155* in the serum of patients with BC and not in healthy controls; this miRNA has been used to monitor the effect of taxane treatment on BC. Sun et al. observed the decreased expression of *Hsa-miR-155* in serum after chemotherapy, which reached levels comparable to those of healthy subjects [144].

#### HIF1 signalling and growth hormone signalling, regulated by *Hsa-miR-490*, *Hsa-miR-429*

Resistance to hormonal therapy is still unknown, but hypoxia could play an important role, for instance, in down-regulating ER-alpha expression as well as ER-alpha function in BC cells [145]. Furthermore, hypoxia and estrogen are interchangeable as both similarly modulate epithelial-endothelial cell interaction [146].

Previous studies showed the role of *Hsa-miR-490* as potential drug resistance in ovarian cancer [147] and as a potential novel biomarker for diagnosing of colorectal cancer [148].

Down regulation of *Hsa-miR-429* was highlighted in the 3D culture-specific miRNA profile better than that in the 2D culture-specific profile, by correlating with the 3D invasive capacity of the MDA-MB-231 BC cell line [149].

*Hsa-miR-429* could be also a regulator of HIF1 Signalling, Glioblastoma Multiforme, Acute Phase Response Signalling and HIF1 Signalling.

#### HIF1 signalling and glioblastoma multiforme signalling regulated by *Hsa-miR-190b*, *Hsa-miR-1246, Hsa-miR-429*

The role of HIF1 Signalling in cancer has already been mentioned above. As regards Glioblastoma multiforme Signalling pathway, among all the altered genes in common with HIF1 Signalling pathway, the main genes are those of the Ras family (i.e., *KRas* and *NRas*), already found mutated in triple-negative BC [150], the genes of the PI3K pathway (i.e., *PIK3C3*, *PIK3CA*), which has been already found silenced or mutated in aggressive BC [151, 152], and those of the serine/threonine protein kinase family, like ATM, already associated with hormone negative early stage BC [153].

As regards miRNAs able to regulate these couples of pathways, we identified *Hsa-miR-429* (already described above), *Hsa-miR-190b* and *Hsa-miR-1246. Hsa-miR-190b* is indicated as a higher discriminating miRNA between ER+ and ER- BC. This miRNA has also an impact on metastasis-free survival and event-free survival rates, independently of ER status [154]. *Hsa-miR-1246* was included in a 5-miRNA signature with good diagnostic features, able to discriminate between healthy and early stage BC samples [155].

#### Acute phase response signalling and HIF1 Signalling regulated by *Hsa-miR-429*, *Hsa-miR-3617*

The role of HIF1 Signalling in cancer has already been mentioned above. Acute Phase Response Signalling has a clear role in both ER+ and triple negative BC [156]. Looking to the genes involved in Acute Phase Response Signalling in common with those of HIF1 Signalling, they belong to the MAP kinase pathway (i.e. *MAPK8*, *MAPK14*) or to the RAS protein family (i.e. *NRas*), as discussed above. *Hsa-miR-429* has already been discussed. As regards *Hsa-miR-3617*, no publication is currently available about the function of this miRNA in any biological processes.

#### Atherosclerosis signalling and acute phase response signalling regulated by *Hsa-miR-1910*

The Acute Phase Response Signalling plays a clear role in BC, as already mentioned. There are only a few publications that associate Atherosclerosis Signalling to BC. However, one of the main molecule involved in tissue remodelling and in atherosclerosis is tenascin-C. Its serum level of expression has no predictive or prognostic ability in BC, although it is elevated in BC patients [157]. As regards miRNAs involved in the control of this couple of pathways, we identified a single miRNA, *Hsa-miR-1910*. This miRNA is included in a group of 8 miRNAs, whose silencing by methylation leads to the onset of BC [158].

## Conclusions

We identified pairwise pathways for BC subtypes able to discriminate BC vs. normal samples. From these pairs, we created a network of pathways specific for each subtype. Following an enrichment analysis, we focused on miRNAs with an important role in the regulation of the network.

In the network of pathways for BC luminal A, we found 11 miRNAs: *Hsa-miR-1-1*, *Hsa-miR-1250*, *Hsa-miR-1537*, *Hsa-miR-205*, *Hsa-miR-210*, *Hsa-miR-3199-1*, *Hsa-miR-335*, *Hsa-miR-337*, *Hsa-miR-381*, *Hsa-miR-452*, and *Hsa-miR-99a*. Among them, *Hsa-miR-210*, and *Hsa-miR-205* have a potential therapeutic role, acting as biomarkers of the response to trastuzumab, and to neoadjuvant chemotherapy, respectively.

In the network of pathways for BC luminal B, we found 3 miRNAs: *Hsa-miR-32*, *Hsa-miR-3074*, and *Hsa-miR-577*. Among them, *Hsa-miR-32* has been already associated with cancer progression.

In the network of pathways for BC basal we found 2 miRNAs: *Hsa-miR-135b*, and *Hsa-miR-365-2*. Among them, *Hsa-miR-365-2* showed an apoptotic role and could play a therapeutic role.

In the network of pathways for HER2 BC, we found 14 miRNAs: *Hsa-miR-1246*, *Hsa-miR-142*, *Hsa-miR-148b*, *Hsa-miR-155*, *Hsa-miR-190*, *Hsa-miR-190b*, *Hsa-miR-1910*, *Hsa-miR-3617*, *Hsa-miR-429*, *Hsa-miR-449c*, *Hsa-miR-490*, *Hsa-miR-511-2*, *Hsa-miR-584*, *Hsa-miR-92b.* Among them, *Hsa-miR-148b*, *Hsa-miR-92b*, *Hsa-miR-142*, *Hsa-miR-155* are interesting for drug design, as a role in the response to different therapeutic strategies has been already described.

The identification of a network of dependent pathways and their regulatory miRNAs is a current challenge in order to have an overview of a complex disease such as cancer. In particular, miRNAs, once validated in a laboratory assay, could be suitable for translation to a clinical environment. The low-cost procedures and the possibility to be measured by non-invasive tests make miRNAs important diagnostic and therapeutic tools for further studies.

## Additional files

Additional file 1: miRNA-r for each pairwise pathway and gene target in luminal A. (DOCX 16 kb)
Additional file 2: miRNA-r for each pairwise pathway and gene target in luminal B. (DOCX 11 kb)
Additional file 3: miRNA-r for each pairwise pathway and gene target in Basal. (DOCX 11 kb)
Additional file 4: miRNA-r for each pairwise pathway and gene target in HER2. (DOCX 16 kb)

## Abbreviations

AUC: Area under the curve
BC: Breast cancer
HER2: Human epidermal growth factor receptor 2
IPA: Ingenuity pathway analysis
MI: Mutual Information
miR-r: miRNAs regulating
NGS: Next generation sequencing
NS: Normal samples
