# Supplementary material for: How interacting pathways are regulated by miRNAs in breast cancer subtypes
Source: BMC Bioinformatics. 2016 Nov 8;17(Suppl 12):111–33. doi: 10.1186/s12859-016-1196-1 (PMC5123339; doi:10.1186/s12859-016-1196-1)
Supplement: Additional file 1: — miRNA-r for each pairwise pathway and gene target in luminal A. (DOCX 16 kb) [file 12859_2016_1196_MOESM1_ESM.docx]

| Pairwise pathways | miRNA-r | Genes a) | Genes b) |
| --- | --- | --- | --- |
| 1.a)Acute Phase Response Signalling;  b) HIF1 Signalling | *Hsa-miR-205* | *AGT, AMBP, CFB, CRABP2, HMOX2, HRAS, IL1R1,MAP3K1, NGFR, PIK3CD, RELA,SERPINF, SOS2, STAT3,*  *TCF4,TRAF6* | *ARNT, EGLN3,HRAS, MMP14 MMP23B, MMP3 MMP9, P4HTM, PIK3C2G, PIK3CD, TCEB1* |
| 2.a)Axonal Guidance Signalling;  b)Acute Phase Response Signalling | *Hsa-miR-205* | *ABL1,ADAMTS7, BMP1, BMP5, BMP7, ECEL1, EFNA4, EPHA2, FZD10, GLI1, GLI2, GLI3, GNB1, HRAS, IGF1, KLC1,*  *LIMK2,MICAL1,MKNK1, MMP9, NGFR,PIK3C2G,PIK3CD,PLCB2, PLXND1,PRKAC, ROBO1, ROBO3, SEMA4A,SEMA5, SOS2, TUBA8, TUBB6, UNC5A* | *AGT, AMBP, CFB, CRABP2, HMOX2, HRAS, IL1R1, MAP3K14, NGFR, PIK3CD, RELA, SERPINF2, SOS2, STAT3, TCF4, TRAF6* |
| 3.a)Axonal Guidance Signalling;  b)Acute Phase Response Signalling | *Hsa-miR-99a* | *ADAM12,ADAM2, ADAMTS1,ADAMTS5, ADAMTS6, AKT3,ARHGEF1, ARHGEF7,ARPC, BMP1, BMP6, BMP7, C9orf3, CXCL12,DPYSL, EFNA5, EFNB1, EPHA3, EPHB1, EPHB4, EPHB6, FZD2, FZD4, FZD7, FZD8, GLI1, GLI2, GLI3, GNAI1, GNAL, GNAZ, GNG11, GNG2, GNG4, IGF1, ITGB1, KIF7, MAPK1, MMP2, MMP7, MRAS, MYL9, NGFR, NRP1, NTRK2, PDGFA, PDGFD, PIK3C2A,PIK3C2B, PLCB4, PLCE1, PLCG2, PLCL2,PRKACB, PRKAG1,PRKCA, PRKCQ, PRKD1, PTCH2, RAC1, RHOA, ROBO1, ROBO3, SDC2, SDCBP,SEMA3A, SEMA3D,SEMA3, SEMA4A,SEMA6, SEMA6D,SLIT2, SLIT3, SMO, SOS1,TUBB2B, WIPF1,WNT10A, WNT3, WNT6* | *A2M, AKT3, C1R, C1S, C3, HP, IL1R1, IL1RAP, IL33, IRAK1, ITIH4, JAK2, JUN, MAP3K14,*  *MAP3K1, MAPK12, MAPK14, MAPK1, MRAS, NGFR,NR3C1,*  *OSMR, RBP4, RBP5, SAA4, SERPINF1, SERPING1, SOCS2, SOCS3, SOS1, STAT3, TCF4,TF, TNFRSF11B,TNFRSF1B, VWF* |
| 4.a)Axonal Guidance Signalling;  b)Acute Phase Response Signalling | *Hsa-miR-335* | *ADAM8,ADAMTS, ARPC5L,EFNB2, EPHA1, FIGF, FZD10, FZD8, GNAL, MAPK3, MMP9, MRAS, NFATC1,NFATC, NTF3,PDGFD, PGF, PIK3R1, PLCD1, PLCG1, PRKAR2B,PRKC, PTPN11,WNT10, WNT6* | *FN1, FOS, HMOX1, HP, IRAK1, MAPK3, MRAS, MYD88, NFKBIE,NR3C1, PIK3R1, PTPN11, RBP4, TF,TNF* |
| 5.a)Axonal Guidance Signalling;  b)Acute Phase Response Signalling | *Hsa-miR-337* | *ACTR3,ADAM12, ADAMTS1,ADAMTS2, ADAMTS5, ADAMTS6, ADAMTS9,AKT3,*  *BMP2, BMP4, BMP6, BMP7, BMP8A, CFL2, CRKL, DPYSL2,*  *EFNB3, FZD1, GLI1, GLI2, GNAL, GNB4, GNG11, GNG4,*  *IGF1, ITGB1, ITSN1, L1CAM, LIMK1, MMP13, MMP2, MYL3,*  *MYL9, NGFR, NRP1, NTRK3, PDGFD, PGF, PIK3C2A,PIK3CA, PIK3CB, PLCB1, PLCB4, PLCL1, PLCL2, PPP3CC,PRKCA, PRKD1, PRKD3, PXN, ROBO1, SDC2, SEMA3C, SEMA3D,SEMA5A, SEMA6B,*  *SEMA6C,SEMA6, SLIT2,TUBA1A, TUBB2A,TUBB2, TUBB6,UNC5C, VEGFC, WNT3, WNT5B* | *A2M, AKT3, C1R, C1S, C5, ECSIT, FN1, IKBKE,IL1R1, IL1RAP, ITIH2, MAPK11, MAPK12, NFKB1, NFKB2, NFKBIB, NGFR, NR3C1, PIK3CA, PIK3CB, RBP2, RIPK1, SERPINF1, SERPING1, SOCS2, SOCS5, TCF4, TNFRSF1A, TRADD* |
| 6.a)Axonal Guidance Signalling;  b)Acute Phase Response Signalling | *Hsa-miR-452* | *ADAMTS2,ADAMTS4, ADAMTS9, ARHGEF15, ARHGEF7, BMP6, CXCL12, EFNB2, EPHA4, EPHB1, EPHB6, FES,FZD4,FZD,*  *GLI2, GNAL, GNB5, GNG2, IGF1, ITSN1, LIMK1, MMP7, MRAS, MYLPF, NFATC1, NRP1, PDGFD, PFN2,*  *PGF, PIK3C2B, PIK3CA,PLCB1, PLCL2, PRKCQ, PTPN11,ROBO3, SEMA6B,SEMA6C, SLIT3, SMO, VEGFB, WNT5B* | *A2M, CRABP2, FN1, HP, IL33, IL6R, MAP2K4, MAP3K1, MAPK12, MRAS, NR3C1, PDPK1, PIK3CA, PTPN11, RBP4, SAA2, SAA4, SOCS3, TF, TNF, VWF* |
| 7.a)Axonal Guidance Signalling;  b)Acute Phase Response Signalling | *Hsa-miR-1250* | *ACTR2, ADAM12 ADAM17,ADAM2, AKT3, ARPC1B, BMP5, ECEL1, EFNA3, EFNB2, EPHB1, EPHB6, FARP2, FZD1, GLI2, GLIS2, GNAI1, GRB2, ITGB1, MYL2, MYL5, MYL7, NFATC3, NGFR, NRP2, PIK3CD, PIK3R2,PLCB1, PLCL1,PLXNA3, PRKCI, PRKCZ, PRKD1, ROBO1, ROCK1,SEMA3A, SEMA4F,SLIT2, SLIT3, WNT4, WNT6* | *AKT3, C1R, GRB2, IKBKE, IL33, IRAK1, ,ITIH2, NGFR, OSM, PIK3CD, PIK3R2, SERPINE1, SERPINF1, SERPINF2,*  *TCF3, TCF4, TTR* |
| 8.a)Ethanol Degradation IV;  b)Glioma Invasiveness Signalling | *Hsa-miR-3199-1* | *ACSL1,ACSS1,ACSS3* | *ITGB5,PIK3C2B, PIK3CB, RHOH, RHOU, VTN* |
| 9.a)Ethanol Degradation IV;  b)Estrogen Receptor Signalling | *Hsa-miR-1-1* | *TYRP1* | *CTBP2, GTF2F2* |
| 10.a)Glioma Invasiveness Signalling;  b)Oxidative Ethanol Degradation III | *Hsa-miR-3199-1* | *ITGB5,PIK3C2, PIK3CB, RHOH, RHOU, VTN* | *ACSL1, ACSS1, ACSS3* |
| 11.a)Intrinsic Prothrombin Activation;  b)Extrinsic Prothrombin Activation | *Hsa-miR-99a* | *COL1A2,COL3A1, F10, F13A1, F5, KLK3,*  *PROS1* | *F10, F13A1, F5, PROS1, TFPI* |
| 12.a)Intrinsic Prothrombin Activation;  b)Extrinsic Prothrombin Activation | *Hsa-miR-210* | *COL10A1, COL1A2, F5,THBD* | *F5, TFPI, THBD* |
| 13.a)Intrinsic Prothrombin Activation;  b)Extrinsic Prothrombin Activation | *Hsa-miR-381* | *COL11A2,COL1A1, COL1A2, COL3A1, F10, F13A1, KLK3, PROS1* | *F10, F13A1, PROS1, TFPI* |
| 14.a)Intrinsic Prothrombin Activation;  b)Extrinsic Prothrombin Activation | *Hsa-miR-1537* | *F12, F13A1, FGB* | *F12, F13A1, FGB* |
