# Supplementary material for: How interacting pathways are regulated by miRNAs in breast cancer subtypes
Source: BMC Bioinformatics. 2016 Nov 8;17(Suppl 12):111–33. doi: 10.1186/s12859-016-1196-1 (PMC5123339; doi:10.1186/s12859-016-1196-1)
Supplement: Additional file 2: — miRNA-r for each pairwise pathway and gene target in luminal B. (DOCX 11 kb) [file 12859_2016_1196_MOESM2_ESM.docx]

| Pairwise pathways | miRNA-r | Genes a) | Genes b) |
| --- | --- | --- | --- |
| 1.a) Epithelial Adherens Junction Signalling;  b) EIF2 Signalling | *Hsa-miR-32* | *ACVR2A, CRK, EGF, HRAS, KEAP1, MYH2, SORBS1, TUBB, TUBG1* | *EIF2AK2, EIF4E, HRAS, PIK3C2G, RPL7, RPL8, RPS12, RPS23, RPS5* |
| 2.a) Epithelial Adherens Junction Signalling;  b) EIF2 Signalling | *Hsa-miR-3074* | *ARPC5L, MRAS, MYH9, NOTCH1, SSX2IP, TCF3* | *EIF2S1, FAU, MRAS, PIK3C3, RPL35* |
| 3.a) Epithelial Adherens Junction Signalling;  b) EIF2 Signalling | *Hsa-miR-577* | *ACTG1, ACTG2, CDC42, DLL1, PTPRM, TGFBR2, TUBB* | *RPL11, RPL14, RPL27, RPL37A, RPL3, RPS24, RPS4Y1* |
