# Supplementary material for: How interacting pathways are regulated by miRNAs in breast cancer subtypes
Source: BMC Bioinformatics. 2016 Nov 8;17(Suppl 12):111–33. doi: 10.1186/s12859-016-1196-1 (PMC5123339; doi:10.1186/s12859-016-1196-1)
Supplement: Additional file 3: — miRNA-r for each pairwise pathway and gene target in Basal. (DOCX 11 kb) [file 12859_2016_1196_MOESM3_ESM.docx]

| Pairwise pathways | miRNA-r | Genes a) | Genes b) |
| --- | --- | --- | --- |
| 1.a)Ethanol Degradation IV;  b) Mismatch Repair in Eukaryotes | *Hsa-miR-135b* | *ACSL1, ACSL3, ALDH1A1,ALDH1B1, ALDH3A2, ALDH4A1* | *FEN1, MSH6, RFC2, RFC4, RPA1, SLC19A1* |
| 2.a)Role of BRCA1 in DNA Damage Response;  b) Putrescine Degradation III | *Hsa-miR-365-2* | *ATR, CHEK2, E2F1, FANCA, FANCB, FANCC, FANCE, MLH1, MSH6, NBN, PLK1, RB1, RBBP8, RBL1, RFC5* | *ALDH1A1,ALDH1A3, ALDH2, IL4I1,*  *MAOA, SMOX* |
| 3.a)Tryptophan Degradation X (Mammalian, via Tryptamine);  b) Role of BRCA1 in DNA Damage Response | *Hsa-miR-365-2* | *ALDH1A1,ALDH1A3, ALDH2, IL4I1, MAOA, SMOX* | *ATR,CHEK2,E2F1,*  *FANCA,FANCB,FANCC, FANCE,MLH1,MSH6, NBN,PLK1, RB1, RBBP8, RBL1,RFC5* |
