# Supplementary material for: How interacting pathways are regulated by miRNAs in breast cancer subtypes
Source: BMC Bioinformatics. 2016 Nov 8;17(Suppl 12):111–33. doi: 10.1186/s12859-016-1196-1 (PMC5123339; doi:10.1186/s12859-016-1196-1)
Supplement: Additional file 4: — miRNA-r for each pairwise pathway and gene target in HER2. (DOCX 16 kb) [file 12859_2016_1196_MOESM4_ESM.docx]

| Pairwise pathways | miRNA-r | Genes a) | Genes b) |
| --- | --- | --- | --- |
| 1.a)Acute Phase Response Signalling;  b) HIF1 Signalling | *Hsa-miR-3617* | *IL6ST,MAPK14, MAPK8,SOCS6,*  *SOCS7, TCF4* | *CREBBP, LDHB, MAPK14, MAPK8, PIK3C3* |
| 2.a)Acute Phase Response Signalling;  b) HIF1 Signalling | *Hsa-miR-429* | *CP, IL18, MAP2K7,NRAS, SERPINA1,*  *SERPING1* | *ATM, CREBBP, NRAS, PIK3C3, VEGFB* |
| 3.a)Atherosclerosis Signalling;  b) Acute Phase Response Signalling | *Hsa-miR-1910* | *ALB, CD40, ITGA4, PLA2G7, RARRES3, TNF* | *AKT1, ALB, HP, MAP2K3, MAP2K4, MAPK3, PDPK1, PIK3R2, TF, TNF* |
| 4.a)Axonal Guidance Signalling;  b) CXCR4 Signalling | *Hsa-miR-148b* | *ADAM10,EFNB1, EFNB3,EPHB1,*  *GNAZ, IGF1,*  *MYL12A,MYL12B, NRAS, PGF,*  *PRKAR1A,PRKCD, STK36,TUBB* | *GNAZ, MYL12A, MYL12B, NRAS, PRKCD, RND2* |
| 5.a)Axonal Guidance Signalling;  b)CXCR4 Signalling | *Hsa-miR-190* | *ADAM8,BMP1,*  *FZD9, GNB2L1,*  *HRAS, NGEF, PIK3C3, PIK3CB, ROCK1, SEMA3E* | *GNB2L1, HRAS, PIK3C3, PIK3CB, ROCK1* |
| 6.a)Axonal Guidance Signalling;  b)P2Y Purigenic Receptor Signalling Pathway | *Hsa-miR-190* | *ADAM8, BMP1,*  *FZD9, GNB2L1,*  *HRAS, NGEF, PIK3C3, PIK3CB, ROCK1, SEMA3E* | *GNB2L1, HRAS, NFKB1, PIK3C3l, PIK3CB* |
| 7.a)Axonal Guidance Signalling;  b)CXCR4 Signalling | *Hsa-miR-449c* | *ADAM19, CXCR4, DPYSL5, ERBB2, FZD2, HKR1, MYL2, NRP2, PPP3R1, PRKCH,*  *PRKD1, ROBO* | *CXCR4, FNBP1, MYL2, PRKCH, PRKD* |
| 8.a)Axonal Guidance Signalling;  b)CXCR4 Signalling | *Hsa-miR-584* | *ARHGEF12, ARPC3,*  *ARHGEF15, ATM, FARP2, GNAI2,*  *GNB2, MAP2K1,*  *NRAS, PLCB2,*  *PLCD3,PXN,*  *TUBA4A, UNC5B,*  *WAS, WIPF1,*  *WNT10A* | *ATM, GNAI2, GNB2, MAP2K1, MAPK12, NRAS, PLCB2, PXN* |
| 9.a)Axonal Guidance Signalling;  b)P2Y Purigenic Receptor Signalling Pathway | *Hsa-miR-584* | *ARHGEF12,*  *ARHGEF15, ARPC3,ATM,*  *FARP2, GNAI2,*  *GNB2, MAP2K1,*  *NRAS, PLCB2,*  *PLCD3, PXN, TUBA4A, UNC5B,*  *WAS, WIPF1,*  *WNT10A* | *ATM, GNAI2, GNB2, MAP2K1, NRAS, PLCB2, PLCD3* |
| 10.a)Axonal Guidance Signalling;  b)P2Y Purigenic Receptor Signalling Pathway | *Hsa-miR-92b* | *CXCL12, EFNB3, FZD5, FZD8,*  *GNB2L1, GNG5,*  *KIF7, LINGO1, MAPK1, PLXNA1, PLXND1, PRKACA, PRKAG1, PRKAR1B, SEMA4B, TUBA1A, TUBB2B* | *GNB2L1, GNG5, MAPK1, PRKACA, PRKAG1, PRKAR1B* |
| 11.a)HIF1 Signalling;  b)Glioblastoma Multiforme Signalling | *Hsa-miR-1246* | *KRAS, MMP26,*  *NRAS, PIK3C2B* | *KRAS, NRAS, PIK3C2B, RHOJ, TSC1* |
| 12.a)HIF1 Signalling;  b)Glioblastoma Multiforme Signalling | *Hsa-miR-190b* | *COPS5, CREBBP, MMP15, MMP24, NOS3, NRAS, PIK3CA* | *FOXO1, FZD8, NRAS, PDGFRB, PIK3CA, PLCL1, RHOH, RHOQ* |
| 13.a)HIF1 Signalling;  b)Glioblastoma Multiforme Signalling | *Hsa-miR-429* | *ATM, CREBBP,*  *NRAS, PIK3C3,*  *VEGFB* | *ATM, CDK2, E2F5, NRAS, PIK3C3, RHOC* |
| 14.a)HIF1 Signalling;  b)Growth Hormone Signalling | *Hsa-miR-429* | *ATM, CREBBP,*  *NRAS, PIK3C3,*  *VEGFB* | *ATM, CDK2, E2F5, NRAS, PIK3C3, RHOC* |
| 15.a)HIF1 Signalling;  b)Growth Hormone Signalling | *Hsa-miR-490* | *EGLN1, EP300,*  *JUN, MDM2,*  *PIK3C2A, PIK3C3* | *MDM2, PIK3C2A, PIK3C3, PLCB4, PLCD3* |
| 16.a)Role of Macrophages, Fibroblasts and Endothelial Cells in Rheumatoid Arthritis;  b)Growth Hormone Signalling | *Hsa-miR-142* | *APC, CCL5, CCND1, FGF2,*  *GNAQ, IL10,*  *IL18R1, IL6R,*  *LTB, MAP3K14, NFATC2, NFATC3, NFKBIE, PIK3CD, PIK3CG, PIK3R5, PIK3R6, PLCB2, PLCG2, PLCL2, PRKCB, PRKCQ,*  *TCF7, TLR10,*  *TLR4, TLR8, TNFRSF1B, TRAF1* | *PIK3CD, PIK3CG, PIK3R5, PIK3R6, PLCG2, PRKCB, PRKCQ, STAT5A, STAT5B* |
| 17.a)Role of Macrophages, Fibroblasts and Endothelial Cells in Rheumatoid Arthritis;  b)Growth Hormone Signalling | *Hsa-miR-155* | *APC, CCL5, CHUK, CREB5,*  *DKK3, GNAQ,*  *IL18R1, IL18, IL1R2, IL1RAP, PDIA3, PIK3CD, PIK3CG,PIK3R5, PLCB2, PLCG2,*  *PPP3CB, PRKCB, PRKCQ, SFRP2, TLR10, TLR6, TLR8,TNFRSF1B,*  *TRAF1* | *IGFALS, PIK3CD, PIK3CG, PIK3R5, PLCG2, PRKCB, PRKCQ, PTPN6, RPS6KA1, STAT1* |
| 18.a)Role of Macrophages, Fibroblasts and Endothelial Cells in Rheumatoid Arthritis;  b)Growth Hormone Signalling | *Hsa-miR-511-2* | *CCL5, CSF2, IL15, IL18R1, IL1R2, IL32, IRAK2, LTB, MAP3K14,MYD88, NFATC2,NFKBIA, NFKBIE,PIK3CD, PIK3CG,PIK3R5, PIK3R6, PLCB2, PLCE1, PLCG2, PRKCB, PRKCH, PRKCQ, SOCS1,*  *SOCS3, TLR10, TLR4, TLR6, TLR8,TNFRSF1B, TNF, TRAF1* | *A2M, PIK3CD, PIK3CG, PIK3R5, PIK3R6, PLCG2, PRKCB, PRKCH, PRKCQ, SOCS1, SOCS3, STAT1, STAT5A* |
